# PregMedNet: Multifaceted maternal medication impacts on neonatal complications

**DOI:** 10.1038/s41467-026-75000-0

**Published:** 2026-07-10

**Authors:** Yeasul Kim, Ivana Marić, Chloe M. Kashiwagi, Lichy Han, Philip Chung, Jonathan D. Reiss, Lindsay D. Butcher, Kaitlin J. Caoili, Eloïse Berson, Lei Xue, Camilo Espinosa, Tomin James, Sayane Shome, Feng Xie, Marc Ghanem, David Seong, Alan L. Chang, S. Momsen Reincke, Samson Mataraso, Chi-Hung Shu, Davide De Francesco, Martin Becker, Wasan M. Kumar, Ronald J. Wong, Brice Gaudilliere, Martin S. Angst, Gary M. Shaw, Brian T. Bateman, David K. Stevenson, Lawrence S. Prince, Nima Aghaeepour

**Affiliations:** 1 Department of Anesthesiology, Perioperative, and Pain Medicine, Stanford School of Medicine, Stanford, CA USA; 2 Department of Pediatrics, Stanford University School of Medicine, Stanford, CA USA; 3 Department of Biomedical Data Science, Stanford University, Stanford, CA USA; 4 Immunology Program, Stanford University School of Medicine, Stanford, CA USA; 5 Mines Paris, PSL Research University, CBIO-Centre for Computational Biology, Paris, France; 6 Institut Curie, PSL Research University, Paris, France; 7 INSERM, U1331, Paris, France; 8 Division of Computational Health Sciences, Department of Surgery, University of Minnesota, Minneapolis, MN USA; 9 Medical Scientist Training Program, Stanford University School of Medicine, Stanford, CA USA; 10 Department of Neurology and Experimental Neurology, Charité—Universitätsmedizin Berlin, Berlin, Germany; 11 Department of Mathematics and Computer Science, Philipps-Universität Marburg, Marburg, Germany; 12 Hessian.AI, Darmstadt University of Technology, Darmstadt, Germany; 13 Medical Doctor Program, Stanford University School of Medicine, Stanford, CA USA; 14 Graduate School of Business, Stanford University School of Medicine, Stanford, CA USA

**Keywords:** Translational research, Paediatric research, Machine learning, Statistical methods

## Abstract

While medication use is common among pregnant women, medication safety remains insufficiently characterized because studies in pregnant women are challenging due to safety concerns. The recent digitization of healthcare databases and advances in computational methods have created new opportunities for large-scale, retrospective drug safety evaluations. Here, we present PregMedNet, a platform that characterizes multifaceted maternal medication associations on neonatal outcomes during pregnancy, covering more than 27,000 drug-disease pairs across 1,152 medications and 24 outcomes. These results encompass known and additional odds ratios (ORs), adjusted ORs, and drug-drug interactions, systematically analyzed using nationwide claims data and an advanced machine learning pipeline. Notably, one of the associations identified in this study is supported by in vivo experiments, increasing confidence in PregMedNet’s findings and highlighting the utility of claims data and machine learning for perinatal medication safety studies. Additionally, potential biological mechanisms underlying the associations are explored using a graph learning method, providing candidate pathways for future mechanistic investigations. We expect that PregMedNet will contribute to advancing maternal medication safety and improving neonatal outcomes by providing extensive, multifaceted drug safety information on this previously underrepresented population.

## Abstract (web summary)

While maternal medication use is widespread, its safety remains incompletely understood. Here, the authors present large-scale, multifaceted associations between maternal medications and neonatal outcomes using claims data and machine learning.

## Introduction

More than 80% of women use at least one medication during pregnancy, and the use of prescription medication has increased by over 60% over the last three decades^1–3^. However, medication safety is still insufficiently characterized in pregnant women, and pregnant women and their healthcare teams frequently encounter limited or contradictory guidance on medication safety^4^. More than one-third of newly approved drugs by the United States (US) Food and Drug Administration (FDA) during the last decade are out of compliance with the Pregnancy and Lactation Labeling Rule (PLLR), and less than 20% have adequate drug safety data on pregnancy and lactation based on human studies^5^.

One of the main reasons for the lack of medication safety evidence in pregnant women is the recognition of pregnant women as a vulnerable population, and regulatory guidance has prioritized decreasing harm. Premarketing clinical trials for drug safety generally pose greater than minimal risk to pregnant women, which conflicts with FDA’s and institutional review board’s priority to provide special protections for pregnant women^6–8^. Because of this, pregnant women have been considered therapeutic orphans due to the lack of adequate drug safety and efficacy data^9–12^. The current drug safety data on pregnancy have been collected mainly through post-marketing surveillance^13^, especially through spontaneous reporting systems (SRSs)^14^. Although SRSs are regarded as an effective tool for drug safety surveillance, they are potentially biased by under-reporting, may not be able to assess low-level risks, and are challenged to definitively quantify risks and side effects^13–16^.

Computational and data-driven approaches to assess drug safety have recently become viable, fueled by rapid digitization of patient data resulting in large healthcare databases, and the development of machine learning^10^. Large administrative claims databases are increasingly being used to assess drug safety because they contain extensive samples of real-world patient information. Unlike electronic health records (EHR), which are typically confined to a single hospital system, claims databases include patients across institutions and geographic regions, thereby providing large enough sample sizes, rich information on potential confounders, and analytic cost-effectiveness. This makes them an invaluable tool for analysis of medication effects, particularly in underrepresented populations such as pregnant women. Previous studies have demonstrated that claims databases can be successfully utilized to evaluate drug safety in pregnant women, by developing algorithms to establish a pregnant cohort and applying statistical analysis^17–20^. However, these studies have typically concentrated on the effects of one or a few maternal medications on pregnancy outcomes.

We introduce PregMedNet, a platform dedicated to advancing maternal medication safety during pregnancy, with particular focus on neonatal complications. The impacts of multiple maternal medications used during pregnancy on neonatal outcomes were assessed based on the Merative™ Marketscan® Commercial Database^21^, a comprehensive collection of claims data across the U.S. covering more than 250 million patients. Utilizing retrospective, large-scale mother-baby dyads, we systematically analyzed the multifaceted maternal medication impacts during pregnancy on neonatal complications with high statistical power.

First, we comprehensively analyzed 27,648 drug-disease pairs between 1152 maternal medications and 24 neonatal complications, encompassing all medications available in the database. To minimize bias, we implemented a machine–learning–based confounding adjustment pipeline, which automatically selects and adjusts for confounding factors without requiring manual selection for each medication-complication pair. Additionally, we analyzed drug–drug interactions (DDIs), identifying additive effects of maternal drug combinations on neonatal complications. In total, 1446 odds ratios (ORs), 261 adjusted ORs, and 5 DDIs were statistically significant, encompassing both known and additional maternal medication associations.

Notably, we experimentally supported one of the associations identified in this study—between maternal ondansetron use and neonatal bronchopulmonary dysplasia (BPD)—in vivo. This experimental support increases confidence in PregMedNet findings and highlights the potential of retrospective claims data and machine learning for maternal medication safety research.

Finally, we employed a knowledge graph and a graph learning method to uncover potential biological mechanisms underlying the identified associations in this study. A case study of ondansetron and BPD demonstrated how this approach generates testable hypotheses, aiming to expedite the mechanism of action (MoA) discovery by narrowing down specific molecular targets for further investigation.

Our comprehensive findings on maternal medication impacts and underlying mechanisms can be explored through an interactive website: https://pregmednet.stanford.edu/.

## Results

### Mother-baby cohort: encompassing a wide range of variables

A total of 1,190,257 mother-baby dyads were obtained between 2007 and 2021 from the Merative™ Marketscan® Commercial Database^21^ (Supplementary Figs. 1 and 2). The average maternal age was 31.18 ± 4.75 years, and the average gestational age was 38.58 ± 1.71 weeks, with a preterm birth rate of 8.98% (Supplementary Data 1). Among newborns, 614,695 (51.6%) were male, and 575,562 (48.4%) were female.

**Fig. 1:**
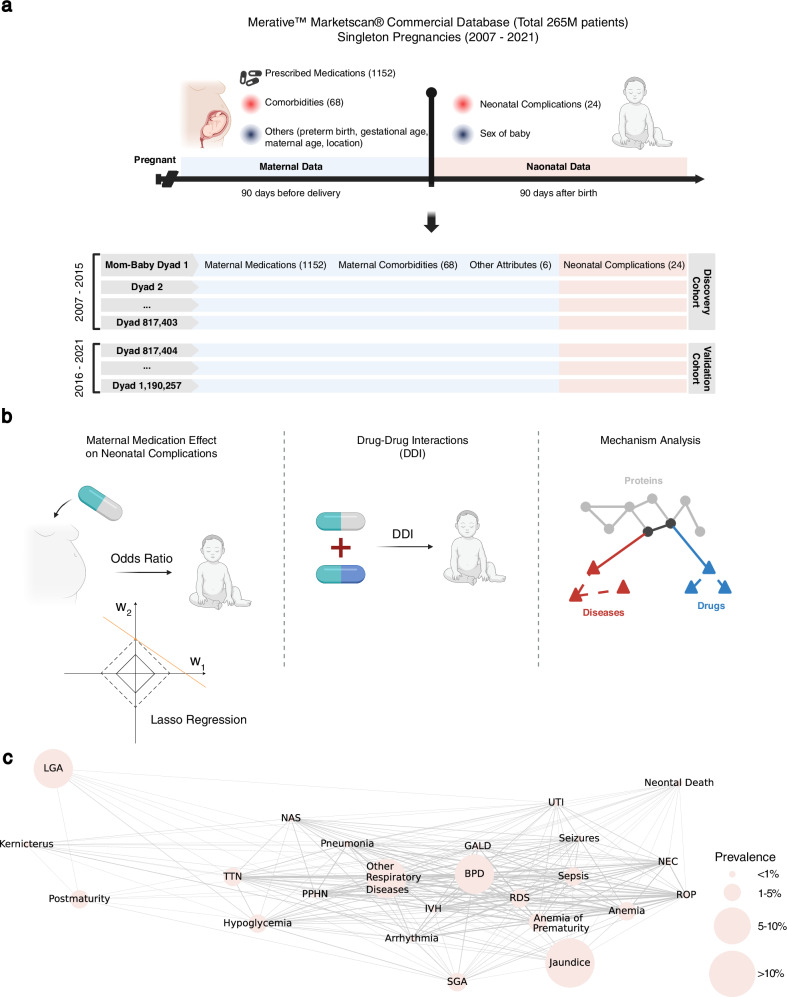
Study overview. **a** A total of 1,190,257 mother-newborn dyads from 2007 to 2021 are extracted from the Merative™ Marketscan® Commercial Database, a set of de-identified claims data accessed through the Stanford Center for Population Health Sciences. Maternal records from the last 90 days before delivery are examined to extract 68 maternal comorbidities, 6 additional attributes, and all prescribed medications. Similarly, newborn records from the first 90 days after birth are analyzed to identify 24 neonatal complications. The final dataset includes 1152 maternal medications, 68 maternal comorbidities, 24 neonatal complications, and 6 additional attributes: maternal age, gestational age at delivery, preterm birth, baby’s sex, and the region and geographic location of the healthcare facility. The dyads are divided into a discovery cohort (2007–2015) and a validation cohort (2016–2021) based on the year of birth. **b** Three experiments are conducted based on the database. First, the associations between maternal medications and neonatal complications are calculated using odds ratios based on logistic regression. Notably, confounders are selected and adjusted for each medication-disease pair using the LassoNoExp algorithm, which penalizes and selects confounding variables using Lasso regression while preserving maternal medication exposure information. Second, the additive effects of concomitant maternal medications on neonatal complications are calculated through drug–drug interaction analysis. Lastly, potential underlying mechanisms of the drug-disease associations are proposed using a knowledge graph and a graph learning method. **c** A tetrachoric correlation plot of the 24 neonatal outcomes shows the relationships between the neonatal complications. The size of each node is correlated with disease prevalence, and the nodes are connected if the diseases are positively correlated. The edge thickness is proportional to the strength of the correlation. Created in BioRender. Kim, Y. (2026) https://BioRender.com/w61v866. Created in BioRender. Kim, Y. (2026) https://BioRender.com/usmpdna.

The discovery cohort included 817,402 dyads, used as the primary group for initial analysis and hypothesis generation. The validation cohort included 372,855 dyads, used to validate the generated hypotheses. Each cohort contained information about pregnant mothers in the 90 days prior to delivery, including 68 maternal comorbidities, preterm birth, gestational age at birth, maternal age at birth, the region and geographic location of the healthcare facilities, and all maternal medications taken during the 90 days prior to delivery. Additionally, the cohort included information about the corresponding babies during the first 90 days after birth, covering both sex and incidence of 24 neonatal complications (Fig. 1.c), with prevalence presented in Supplementary Data 2. All maternal medications taken were extracted and harmonized to the medications’ compound-level identity, along with route of administration information (Supplementary Fig. 3). There were a total of 1152 maternal medications in the discovery cohort and 980 in the validation cohort.

### Multifaceted insights into perinatal medication safeties

PregMedNet offers comprehensive insights into the associations between maternal medications and neonatal complications, examining (a) effects of single medications, (b) impacts of concomitant medications on neonatal outcomes, and (c) potential underlying mechanisms behind significant associations.

### Individual maternal medication impacts on newborn outcomes

The associations between maternal medications and neonatal complications were investigated by estimating both unadjusted and adjusted odds ratios (ORs and aORs, respectively). Out of 27,648 medication-complication pairs, 1446 ORs (5.23%) and 261 aORs (0.94%) were statistically significant, encompassing both already known and additional maternal medication associations with neonatal complications.

### Network graphs visualize intricate patterns of associations

**Fig. 2:**
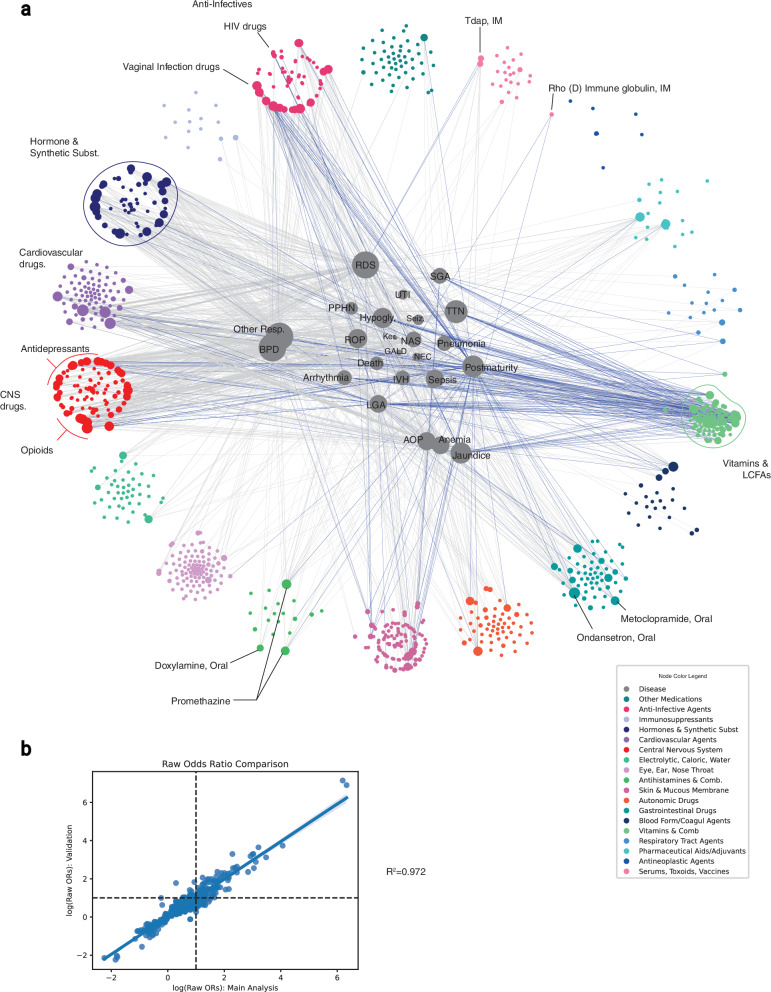
Network of associations between maternal medications and neonatal complications with cohort-based replication. **a** Odds ratio (OR) results are displayed as a network graph, where nodes represent maternal medications or neonatal complications, and edges indicate statistically significant ORs. Disease nodes are centrally placed, while maternal medication nodes are peripheral. Medications are grouped and color-coded within each cluster. The graph shows significant ORs as edges; gray for ORs above 1 (positive correlations), and blue for ORs below 1 (negative correlations). There are 24 disease nodes, 1152 medication nodes, and 1446 edges—1153 gray and 293 blue. Edge thickness correlates with the strength of the association (inverse of *p*-values), and node size with the number of connecting edges. Abbreviations in disease nodes: BPD bronchopulmonary dysplasia, Other Resp. other respiratory diseases, RDS respiratory distress syndrome, SGA small for gestational age, PPHN persistent pulmonary hypertension, Hypogly. hypoglycemia, UTI urinary tract infection, Seiz. seizures, TTN transient tachypnea of the newborn, ROP retinopathy of prematurity, Ker. kernicterus, GALD gestational alloimmune liver disease, NAS neonatal abstinence syndrome, NEC necrotizing enterocolitis, IVH intraventricular hemorrhage, Death neonatal death, LGA large for gestational age, AOP anemia of prematurity. Additional conditions in disease nodes not abbreviated: Pneumonia, Postmaturity, Sepsis, Arrhythmia, Anemia, and Jaundice. **b** ORs from the discovery cohort were replicated in the validation cohort. ORs for both cohorts were matched by maternal medications and neonatal complications and plotted on a scatterplot. This figure compares the log of significant ORs from the discovery (*x*-axis) to the validation cohort (*y*-axis). Of the ORs, 772 matched between the cohorts—1446 significant in the discovery cohort and 1097 in the validation cohort. Pearson correlation analysis was performed using a two-sided test, yielding an *R*² of 0.972 with a two-sided *p*-value < 1 × 10^-300^.

PregMedNet illustrates these associations using network graphs, with ORs presented in Fig. 2 and aORs in Supplementary Fig. 4. Medications and diseases are represented as nodes clustered by groups, with edges color-coded to indicate increased risk (gray, OR or aOR>1) or reduced risk (blue, OR or aOR<1) associations. The network graphs highlight intricate patterns between medications and diseases.

In the OR network graph (Fig. 2), 1153 edges (79.7%) are gray, while 293 edges (20.3%) are blue, out of a total of 1446 edges. Focusing on diseases, neonatal respiratory diseases, particularly Respiratory Distress Syndrome (RDS) (140 gray edges) and BPD (135 gray edges), exhibit the most increased risk associations with maternal medications. In contrast, Postmaturity (87 blue edges) and Large for Gestational Age (49 blue edges) show the most reduced risk associations. Focusing on maternal medication groups, the Central Nervous System (CNS) group has the highest number of increased risk associations (300 gray edges), followed by the Hormone and Synthetic Substances group (212 gray edges). Conversely, the Vitamins & Combinations group shows the highest number of reduced risk associations (151 blue edges), followed by the Anti-Infective Agents group (40 blue edges). Supplementary Fig. 5 provides detailed information on the number of significant ORs per neonatal complication and maternal medication group.

In contrast, the aOR network graph (Supplementary Fig. 4) reveals a shift toward decreased risk associations, with 117 edges (44.8%) gray and 144 edges (55.2%) blue out of 261 total edges.

**Fig. 3:**
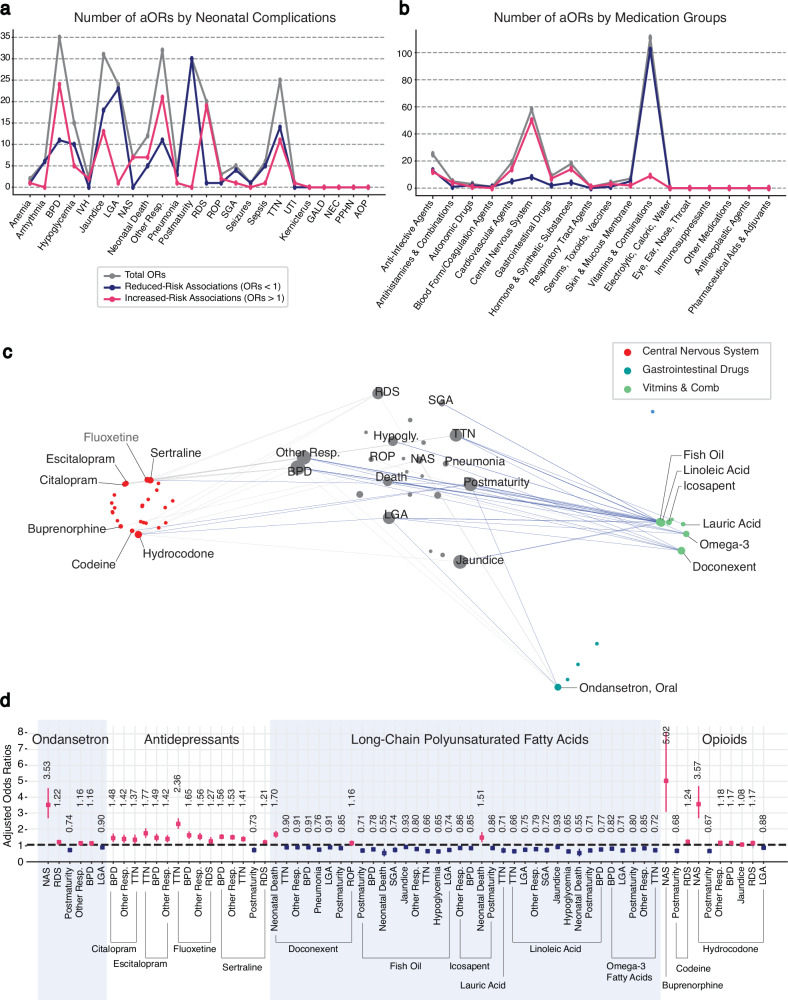
Associations between four maternal medication groups and neonatal complications: highlights from the adjusted odds ratio network. **a** The figure shows the number of significant adjusted odds ratios (aORs) by neonatal complications. The gray line represents the total aORs, the blue line indicates reduced-risk associations (aORs <1), whereas the magenta line indicates increased-risk associations (aORs >1). **b** The figure shows the number of significant aORs by maternal medication groups, with the line colors corresponding to those in (**a**). **c** This network graph highlights four maternal medication groups and their aORs with neonatal complications. Nodes are consistent with those in Fig. 2’s unadjusted odds ratio graph, while edges represent statistically significant aORs. LassoNoExp was utilized to calculate these aORs, selecting and adjusting for maternal covariates from a pool of 73 candidate variables for each drug-complication pair. Edges in gray indicate increased-risk associations (aORs > 1), whereas edges in blue indicate reduced-risk associations (aORs < 1). Edge thickness corresponds to the association strength, inversely proportional to the *p*-values. Highlighted medication groups include Ondansetron, Antidepressants, Long-Chain Polyunsaturated Fatty Acids (LC-PUFAs), and Opioids. The full network can be examined in Supplementary Fig. 6. **d** The subfigure details the values of statistically significant aORs and their 95% confidence intervals (95% CI) for the four medication groups. The results are derived from the discovery cohort of *n* = 817,402 mother–newborn dyads retrieved and processed from the Merative™ Marketscan® Commercial Database. Each dyad represents a single, independent unit of observation. Each dot represents an aOR, while error bars represent 95% CI. Magenta dots and lines depict aORs greater than 1, representing increased-risk associations, whereas blue dots and lines indicate aORs less than 1, representing reduced-risk associations. Numbers on each dot represent the corresponding aOR values. Exact numerical values, including 95% CI and *p*-values are provided in Table 1 and pregmednet.stanford.edu.

Figure 3.a, b present the numbers of significant aORs across neonatal complications and maternal medication groups, respectively, as line charts. In these panels, the gray line represents the total number of significant aORs, whereas magenta and blue lines denote increased-risk (aOR>1) and decreased-risk (aOR<1) associations per each group, respectively. This color scheme differs from that used in the network visualizations (Figs. 2 and 3.c; Supplementary Fig. 4), where gray and blue edges denote increased-risk and decreased-risk associations, respectively.

Focusing on diseases (Fig. 3a), patterns with aORs align with ORs: neonatal respiratory complications show the highest increased risk associations, led by BPD (24 magenta edges), followed by Other Respiratory (21 magenta edges) and RDS (19 magenta edges), while Postmaturity (30 blue edges) and Large for Gestational Age (LGA) (23 blue edges) have the most decreased risk associations. Similarly, focusing on maternal medication groups (Fig. 3b), CNS medications show the highest increased risk associations (50 magenta edges), followed by Cardiovascular Agents and Hormone and Synthetic Substances (14 magenta edges for each), while Vitamins & Combinations show the most decreased risk associations (102 blue edges), followed by Anti-Infective Agents (13 blue edges). Supplementary Fig. 6 provides a detailed quantitative breakdown of the number of aORs per each group—total, increased-risk (aOR > 1), and decreased-risk (aOR <1)—presented as bar charts, complementing the trend-based visualization in Fig. 3a, b.

### Detailed information on associations: highlighted examples of ORs

PregMedNet provides detailed ORs and aORs for all medication-disease pairs through an interactive website, presenting both significant and non-significant results. Supplementary Fig. 7 highlights key examples among 1446 significant ORs.

Notably, within the CNS category, antidepressants and opioids are associated with elevated ORs for several neonatal complications, such as neonatal respiratory disorders and neonatal abstinence syndrome (NAS) (Supplementary Fig. 7.a, b). Similarly, antiemetics—commonly used during pregnancy^22^—are associated with elevated ORs for multiple neonatal complications (Supplementary Fig. 7.c). In contrast, long-chain polyunsaturated fatty acids (LC-PUFAs) in the Vitamins & Combinations category are highlighted here, showing substantially reduced risk associations for multiple neonatal complications, including neonatal respiratory disorders (Supplementary Fig. 7.d). Medications in the Anti-Infective Agents category also exhibit reduced risk associations (Supplementary Fig. 7.e), with oral valacyclovir linked to reduced risks across multiple neonatal complications, consistent with prior studies^17^.

### aORs derived through machine-learning-based confounder adjustment

While ORs provide valuable insights, they are unadjusted for potential confounders, which could introduce bias or spurious associations. In PregMedNet, a machine-learning-based method that selects and adjusts for confounding variables across multiple disease-medication pairs was employed, systematically estimating aORs. In the following section, we focus on four medication groups: ondansetron, antidepressants, LC-PUFAs, and opioids.

### Ondansetron linked to increased neonatal respiratory risks

In PregMedNet, ondansetron is categorized into three types based on the route of administration: oral, oromucosal, and injection. Among these, orally administered ondansetron exhibits six statistically significant aORs, including three elevated risk associations for neonatal respiratory complications: RDS (aOR 1.22, 95% CI 1.13–1.32), BPD (aOR 1.16, 95% CI 1.09–1.23), and other respiratory diseases (aOR 1.16, 95% CI 1.09–1.23). Comprehensive estimates are presented in Table 1 and Fig. 3c, d.

**Table 1:** Adjusted odds ratios (aORs) for four medication groups

Medication Group	Medication	Neonatal Complications	Adjusted odds ratios aOR (95% CI)	*p*
Ondansetron	Ondansetron, Oral	NAS	3.53 (2.75–4.53)	4.19E-23
		RDS	1.22 (1.13– 1.32)	2.17E-07
		BPD	1.16 (1.09–1.23)	1.81E-09
		Other respiratory diseases	1.16 (1.09–1.23)	9.62E-07
		Postmaturity	0.74 (0.65–0.83)	8.81E-08
		LGA	0.90 (0.84–0.97)	2.85E-03
SSRIs	Citalopram, Oral	BPD	1.48 (1.27–1.71)	2.39E-07
		Other respiratory diseases	1.42 (1.22–1.65)	4.72E-06
		TTN	1.37 (1.15–1.65)	5.37E-04
	Escitalopram, Oral	BPD	1.49 (1.32–1.70)	7.36E-10
		Other respiratory diseases	1.42 (1.25–1.62)	1.00E-07
		TTN	1.77 (1.54– 2.04)	2.37E-15
	Fluoxetine, Oral	BPD	1.65 (1.47–1.85)	1.39E-17
		Other respiratory diseases	1.56 (1.39–1.75)	9.62E-14
		TTN	2.36 (2.09–2.65)	5.55E-45
		RDS	1.27 (1.08–1.50)	3.45E-03
	Sertraline, Oral	BPD	1.56 (1.45–1.67)	6.88E-34
		Other respiratory diseases	1.53 (1.42–1.64)	1.66E-30
		TTN	1.41 (1.30–1.54)	5.60E-15
		RDS	1.21 (1.09–1.34)	2.75E-04
		Postmaturity	0.73 (0.63–0.86)	1.04E-04
LC-PUFAs	Doconexent, Oral	Neonatal Death	1.70 (1.54–1.88)	1.45E-25
		TTN	0.90 (0.86–0.94)	1.13E-05
		Other respiratory diseases	0.91 (0.88–0.94)	1.06E-06
		BPD	0.91 (0.87–0.94)	3.35E-07
		Pneumonia	0.76 (0.69–0.84)	2.10E-07
		LGA	0.91 (0.88–0.94)	6.29E-08
		Postmaturity	0.85 (0.81–0.90)	3.07E-08
		ROP	1.16 (1.05–1.28)	0.0032
	Fish Oil, Oral	Postmaturity	0.71 (0.62–0.81)	3.64E-07
		BPD	0.78 (0.71–0.84)	4.18E-09
		Neonatal Death	0.55 (0.39–0.78)	7.05E-04
		SGA	0.74 (0.63–0.87)	2.17E-04
		Jaundice	0.93 (0.89–0.96)	6.41E-05
		Other respiratory diseases	0.80 (0.73–0.87)	1.18E-07
		TTN	0.66 (0.56–0.74)	1.17E-09
		Hypoglycemia	0.65 (0.56–0.74)	1.17E-09
		LGA	0.74 (0.63–0.87)	2.17E-04
	Icosapent, Oral	Other respiratory diseases	0.86 (0.81–0.92)	2.16E-05
		BPD	0.85 (0.79–0.91)	3.23E-06
		Neonatal Death	1.51 (1.27–1.80)	4.55E-06
		Postmaturity	0.86 (0.78–0.95)	0.0030
	Lauric acid, Oral	TTN	0.71 (0.59–0.85)	2.10E-04
	Linoleic acid, Oral	TTN	0.66 (0.59–0.74)	3.39E-13
		LGA	0.75 (0.70–0.82)	3.69E-12
		Other respiratory diseases	0.79 (0.73–0.86)	4.76E-08
		SGA	0.73 (0.62–0.86)	1.15E-04
		Jaundice	0.93 (0.90–0.97)	1.29E-04
		Hypoglycemia	0.65 (0.56–0.74)	1.09E-09
		Neonatal Death	0.55 (0.39–0.78)	7.34E-04
		Postmaturity	0.71 (0.62–0.81)	4.27E-07
		BPD	0.77 (0.71–0.84)	1.82E-09
	Omega-3 fatty acids, Oral	BPD	0.82 (0.76–0.90)	6.28E-06
		LGA	0.71 (0.65–0.77)	1.09E-15
		Postmaturity	0.76 (0.67–0.86)	2.56E-05
		Other respiratory diseases	0.85 (0.78–0.92)	1.23E-04
		TTN	0.72 (0.65–0.80)	5.54E-09
Opioids	Buprenorphine, Sublingual	NAS	5.02 (3.14–8.01)	1.34E-11
	Codeine, Oral	Postmaturity	0.68 (0.59–0.79)	3.56E-07
		RDS	1.24 (1.13–1.36)	2.31E-06
	Hydrocodone, Oral	NAS	3.57 (2.75–4.65)	2.33E-21
		Postmaturity	0.67 (0.58–0.78)	7.05E-08
		Other respiratory diseases	1.18 (1.10–1.26)	7.58E-07
		BPD	1.17 (1.10–1.25)	1.37E-06
		Jaundice	1.08 (1.04–1.11)	4.23E-05
		RDS	1.17 (1.07–1.27)	2.83E-04
		LGA	0.88 (0.82–0.95)	5.26E-04

This table presents selected aORs from four medication groups highlighted in the “Results” section. The results are derived from LassoNoExp applied to the discovery cohort (*n* = 817,402 mother–newborn dyads). Statistical significance was assessed using two-sided tests, and *p*-values were adjusted for multiple comparisons using the Benjamini–Hochberg method. The complete set of 261 significant associations is available on the interactive website (http://pregmednet.stanford.edu).

*SSRIs* selective serotonin reuptake inhibitors, *LC-PUFAs* long-chain polyunsaturated fatty acids, *NAS* NEonatal Abstinence Syndrome, *RDS* respiratory distress syndrome, *BPD* bronchopulmonary dysplasia, *Other respiratory diseases* Other respiratory diseases, *Postmaturity* Postmaturity, *LGA* large for gestational age, *Neonatal Death* neonatal death, *Pneumonia* pneumonia, *ROP* retinopathy of prematurity, *SGA* small for gestational age, *Jaundice* Jaundice, *Hypoglycemia* hypoglycemia.

Ondansetron is a medication used to treat nausea and vomiting during pregnancy, with a prevalence exceeding 25% in the US^23^. The American College of Obstetricians and Gynecologists (ACOG) recommends cautious use of ondansetron during pregnancy due to insufficient safety data, as current studies are limited by small sample sizes and potential recall bias^24^. The European Medicines Agency’s Pharmacovigilance Risk Assessment Committee (EMA PRAC) advises against the use of ondansetron during the first trimester of pregnancy due to recent studies that have found some associations with congenital malformations^25,26^. Our study, based on a large-scale retrospective database, addresses some of these limitations and finds that maternal use of ondansetron during the last 90 days before delivery is associated with neonatal complications, particularly those related to the neonatal respiratory system.

### Neonatal respiratory risks associated with perinatal SSRI use

Four SSRIs—citalopram, escitalopram, fluoxetine, and sertraline, all administered orally—show increased associations with neonatal respiratory complications. For example, citalopram presented increased associations with BPD (aOR 1.48, 95% CI 1.27–1.71), other respiratory disorders (aOR 1.42, 95% CI 1.22–1.65), and transient tachypnea of newborn [TTN] (aOR 1.37, 95% CI 1.15–1.65). Similar associations were observed for the other SSRIs, as detailed in Table 1 and Fig. 3c, d, and these findings are consistent with previous research results^27,28^.

Approximately 10% of pregnant women experience depression, and 2%–3% take antidepressants, yet their use during pregnancy is controversial due to potential adverse effects on neonates^29^. Selective serotonin reuptake inhibitors (SSRIs) are the most commonly used antidepressants during pregnancy, because they have a more favorable safety profile than other antidepressant drug classes. However, findings associate them with neonatal complications^29–31^, including an increased risk of respiratory complications in neonates^27,28^. PregMedNet reconfirms the increased risk of neonatal respiratory complications in perinatal SSRI-exposed babies based on aORs. Specifically, PregMedNet is able to identify associations at the level of individual medications rather than SSRI medications as a whole, thus providing more granular safety information for each medication.

### LC-PUFAs linked to reduced risks across neonatal outcomes

LC-PUFAs, categorized under the Vitamins & Combinations group, exhibited prominent reduced-risk associations with neonatal respiratory complications (e.g., BPD, other respiratory diseases, and TTN), with five out of the six LC-PUFAs identified in the adjusted analyses demonstrating reduced-risk effects.

Fish oil and linoleic acid are particularly noteworthy for their reduced risk associations, each displaying nine reduced risk aORs related to various neonatal complications, including disorders of the respiratory system (BPD, other respiratory diseases, TTN), gestational age or size-related disorders (both LGA and SGA, postmaturity), hypoglycemia, jaundice, and neonatal death. For instance, linoleic acid’s reduced risk associations include BPD (aOR 0.77, 95% CI 0.71–0.84), LGA (aOR 0.75, 95% CI 0.70–0.82), hypoglycemia (aOR 0.65, 95% CI 0.56–0.74), and neonatal death (aOR 0.55, 95% CI 0.39–0.78). Fish oil showed a comparable pattern, with nine reductions across the same domain; the remaining results are provided in Table 1 and Fig. 3c, d.

Previous studies have shown the associations between maternal LC-PUFAs levels during pregnancy and maternal health, including preeclampsia and postpartum depression^32,33^, as well as their association with children’s health, including neurodevelopmental outcomes^34^. Furthermore, recent research suggests that LC-PUFAs may also decrease the incidence of asthma and wheezing in infants, although this evidence remains limited^35^. PregMedNet underscores the reduced risk associations between LC-PUFAs and neonatal respiratory health, aligned with previous findings, while noting previously unrecognized associations with hypoglycemia and neonatal death.

### Opioid use is associated with multiple neonatal complications

As many as 6.6% of pregnant women use prescription opioids, and there is concern that these opioids can cause neonatal complications^36^. PregMedNet found associations between opioids and increased risks of serious neonatal complications. For example, maternal oral hydrocodone exhibited seven increased risk associations, including NAS (aOR 3.57, 95% CI 2.75–4.65) and BPD (aOR 1.17, 95% CI 1.10–1.25), with further outcomes detailed in Table 1 and Fig. 3c, d.

The medications highlighted in this section identified both previously reported and additional associations as ORs and aORs, suggesting the utility of PregMedNet in capturing the associations between maternal medications and neonatal complications. While this section highlights selected medication groups, comprehensive results, including all 1446 ORs and 261 aORs, are available on our interactive website (http://pregmednet.stanford.edu/).

### Systematic analysis of maternal drug–drug interactions

**Fig. 4:**
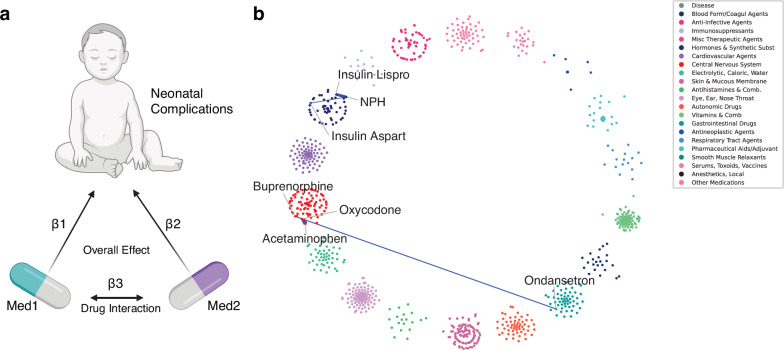
Maternal drug–drug interaction effects on neonatal complications. **a** Drug–drug interaction (DDI) effect from the concomitant use of maternal medications was calculated based on mother-baby dyads. Here, *β*_*1*_ and *β*_*2*_ represent the individual contributions of each medication, while *β*_*3*_ represents the interaction term between the two medications. *β*_*3*_ captures deviations from the expected joint effect of two medications on neonatal complications. **b** A network graph illustrates the DDIs as edges connecting the medication nodes. Notably, five DDIs significant in both discovery and validation cohorts are illustrated. All five edges are colored blue, indicating a downward shift in the odds of neonatal complications compared to the expected joint effects predicted by the individual medications. Created in BioRender. Kim, Y. (2026) https://BioRender.com/iigtmzk.

PregMedNet also captures the additive effects of concurrent use of maternal medications on neonates through a systematic analysis of drug–drug interactions (DDIs) for all possible maternal medication pairs out of 1152 medications and their associations with 24 neonatal complications. $O{R}_{1}$, $O{R}_{2}$, and $O{R}_{12}$ represent the ORs of medication 1, medication 2, and the concurrent use of two medications, respectively, while ${\beta }_{3}$ quantifies the extent to which the DDI modifies the odds of neonatal complications. A negative ${\beta }_{3}$ indicates a downward shift in the odds associated with the interaction, whereas a positive ${\beta }_{3}$ reflects an upward shift. The magnitude of association is proportional to the absolute value of ${\beta }_{3}$. (Fig. 4)

A total of 72 significant DDIs were identified in the discovery cohort and 15 in the validation cohort with 5 DDIs demonstrating significant ${\beta }_{3}$ and $O{R}_{12}$ values in both the discovery and validation cohorts. All five medication pairs exhibit negative ${\beta }_{3}$ values, indicating that the total effect of use of two medications is less than the additive effects of two medications (Table 2).

**Table 2:** Maternal drug–drug interaction effects on neonatal complications

Disease	Medication 1	Medication 2	${\beta }_{3}$	${{{\rm{O}}}}{{{{\rm{R}}}}}_{1}$	${{{\rm{O}}}}{{{{\rm{R}}}}}_{2}$	${{{\rm{O}}}}{{{{\rm{R}}}}}_{12}$
NAS	Acetaminophen, Oral	Oxycodone, Oral	−3.50	4.89	99.97	14.77
Hypoglycemia	NPH, subcutaneous	Insulin Lispro, Injection	−1.84	2.19	5.92	2.07
Hypoglycemia	NPH, subcutaneous	Insulin Aspart, Injection	−1.52	2.14	5.42	2.55
NAS	Buprenorphine, sublingual	Ondansetron, Oral	−1.89	607.34	5.17	474.43
RDS	NPH, subcutaneous	Insulin Aspart, Injection	−1.02	1.44	2.25	1.17

This table details the five significant drug–drug interactions (DDI). The term ${\beta }_{3}$ represents the contribution of the DDI to the overall effect of medication pairs on a neonatal complication. ${{{\rm{O}}}}{{{{\rm{R}}}}}_{1}$, ${{{\rm{O}}}}{{{{\rm{R}}}}}_{2}$, and ${{{\rm{O}}}}{{{{\rm{R}}}}}_{12}$ represent the effect of medication 1, medication 2, and the concurrent use of two medications on a neonatal complication, respectively. A negative ${\beta }_{3}$ indicates the reduced risk effects of the DDI on neonatal complications.

One of the examples is the concurrent use of oral oxycodone and oral acetaminophen. The DDI effect for this combination on NAS was ${\beta }_{3}$ = -3.50, with ${{OR}}_{12}$ = 14.77, which was lower than the risk obtained by adding the effects of each medication alone (oxycodone: $O{R}_{1}$ = 99.97, acetaminophen: $O{R}_{2}=$4.89). This attenuation indicates that concurrent use may modify neonatal risk profiles through DDI. Prior research has shown that combining oxycodone with other analgesics–NSAIDs– improves pain management over oxycodone alone^37^. In a similar manner, our findings highlight the potential implications of oxycodone in combination with other analgesics in the perinatal population.

Another medication pair to highlight is the concurrent use of maternal insulins. The DDI effect of combined use of maternal Insulin Human Isophane (NPH, subcutaneous) and Insulin lispro (injection) for neonatal hypoglycemia is ${\beta }_{3}=$ −1.84, and the $O{R}_{12}$ = 2.03. Notably, the $O{R}_{12}$ is lower than the OR for every single medication (NPH: 2.19, Insulin lispro: 5.97), indicating that the concurrent use is associated with lower odds of neonatal hypoglycemia than predicted by their individual odds. Similarly, the DDI effect of the combination of NPH (subcutaneous) and Insulin aspart (injection) on hypoglycemia is ${\beta }_{3}$ = -1.52 with $O{R}_{12}=$ 2.55, which is lower than the odds observed for Insulin Aspart alone (NPH: $O{R}_{1}=$2.14, Insulin Aspart: $O{R}_{2}=$5.42). These findings are consistent with existing clinical understanding that basal-bolus insulin regimens are commonly used in the management of gestational diabetes (GDM)^38^. Improved glucose regulation minimizes in-utero fetal hyperinsulinemia, a common complication in GDM, potentially correlating with a reduced likelihood of neonatal hypoglycemia at birth.

Lastly, there are two additional medication pairs which show significant ${\beta }_{3}$ in both the discovery cohort and the validation cohort: Buprenorphine (sublingual) and ondansetron (oral) for NAS (${\beta }_{3}$ = -1.89), and NPH (subcutaneous) and Insulin aspart (injection) for RDS (${\beta }_{3}$ = -1.02).

The DDI analysis conducted through PregMedNet shows that certain well-known medication pairs exhibit potentially favorable synergistic associations for neonatal complications when used concurrently in mothers. Although we have spotlighted five medication pairs with overlapping significance in both the discovery and validation cohorts, it is important to note that DDIs identified exclusively in one cohort still hold value. Further details on the additional DDIs are available on our interactive website.

### Reproducibility of single and concomitant medication effects

The ORs and aORs, along with DDI results from the discovery cohorts, were replicated in the independent validation cohort using the same analysis. Scatter plots and Pearson correlation coefficients (Fig. 2b and Supplementary Fig. 8) demonstrated strong consistency (${R}^{2}$ = 0.972 for ORs, ${R}^{2}$ = 0.747 for aORs, and ${R}^{2}$ = 0.920 for DDIs). These results indicate maternal medication associations derived from the discovery cohort were reproducible in the independent cohort across differing conditions, such as study year, suggesting the robustness of PregMedNet findings.

### Maternal ondansetron impairs neonatal lung development in vivo

To further evaluate the robustness of the associations identified in this study, we experimentally investigate the previously unreported association, the effect of maternal ondansetron on fetal and neonatal lung development in vivo. Ondansetron was of particular interest due to its high usage during pregnancy and uncertain perinatal safety^24,25^. Our analysis showed that maternal ondansetron use was associated with increased odds of neonatal BPD (aOR = 1.16, *p*-value = 1.18e-6), warranting further investigation.

A total of 20 female and 10 male C57BL/6 J mice were used for timed mating, from which 9 pregnant dams were included (4 control and 5 ondansetron-treated). Pregnant dams were dosed daily with 5 mg/kg bodyweight ondansetron by intraperitoneal injection from embryonic day 15 (E15) until delivery. This yielded 20 control pups and 24 ondansetron-exposed pups. Following the lung selection and subsequent quality control of lung specimens, 9 controls and 12 ondansetron-exposed pups were included in the final quantitative analysis of the mean linear intercept (MLI). Sex of the pups was not considered in the study design because neonatal mice were used in the study, before sex could be accurately determined.

**Fig. 5:**
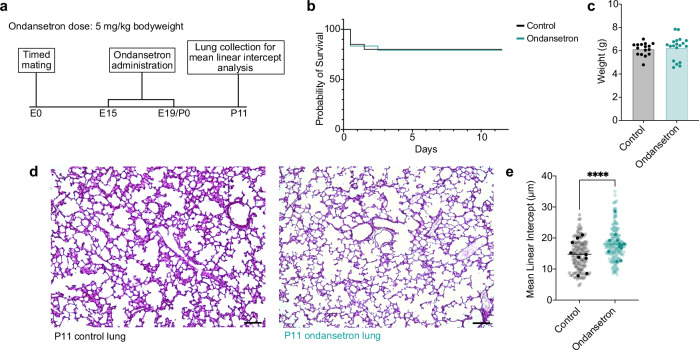
Impact of maternal ondansetron exposure and neonatal lung development in mice. **a** Experimental timeline of maternal ondansetron administration and quantification of lung development. Timed pregnant C57BL/6 J female mice were injected daily with ondansetron (5 mg/kg i.p.) or an equivalent volume of sterile PBS (control) from E15 until delivery. **b** Kaplan–Meier survival analysis and **c** neonatal weight of control and ondansetron-exposed mice. *N* = 20 mice from 4 litters in the control group; *n* = 24 mice from 5 litters in the ondansetron group. **d** Representative H&E images of lungs from control and ondansetron-exposed mice at postnatal day 11. Scale bar, 100 μm. **e** Mean linear intercept quantification of control (9 animals) and ondansetron-exposed mice (12 animals). For each mouse, 3 lung sections were stained, with 5 random images taken from each section. *N* = 135 for controls, *n* = 180 for ondansetron-treated animals. Darker, overlayed dots represent average mean linear intercept values for each individual mouse. **** indicates *p* < 0.0001 as determined using an unpaired, two-tailed *t*-test.

Lung histology was assessed on postnatal day 11 (P11) to test if ondansetron exposure might impact saccular and alveolar stage lung development (Fig. 5). There were no statistically significant differences in reabsorption or litter size, and pregnant dams dosed with ondansetron showed no clinical signs of morbidity. Additionally, maternal ondansetron exposure did not affect neonatal survival or weight gain (Fig. 5b, c), suggesting that secondary influences on lung development can be excluded. However, the lungs of ondansetron-exposed mice had increased alveolar mean linear intercept, indicative of reduced alveolar septation and interstitial septal thinning^39^ (Fig. 5d, e). These findings support an association between maternal ondansetron exposure and subsequent neonatal lung abnormalities, consistent with the associations identified through PregMedNet.

### Knowledge graph reveals potential underlying mechanisms

PregMedNet proposes potential underlying mechanisms of the associations identified in this study between maternal medications and neonatal complications. This was achieved by combining a knowledge graph^40^ and subsequently applying a graph learning method for the 261 significant aORs, which drew associations between 19 neonatal complications and 68 maternal medications. Of these, 15 neonatal complications and 63 maternal medications were mapped from PregMedNet to corresponding nodes in the knowledge graph, resulting in 153 disease-medication pairs for which mechanistic hypotheses are generated.

Two types of subgraphs were constructed: one containing all biological interactions and the other one with only proteins/genes between the medication and disease pair. Hypothesis generation involved extracting the shortest paths between the medication and the disease nodes and examining the involved biological nodes on these paths. This process was iterated across all 153 associations.

**Fig. 6:**
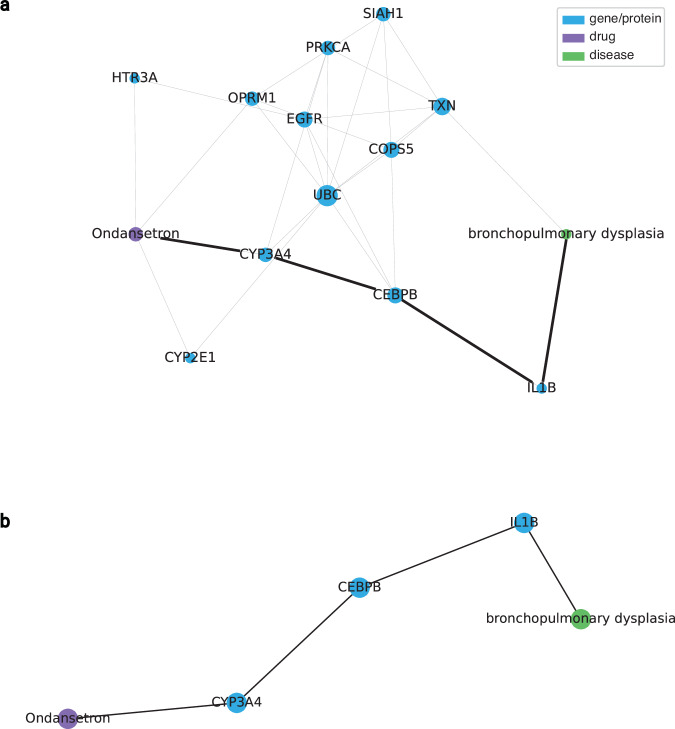
Potential underlying mechanisms of ondansetron effects on bronchopulmonary dysplasia. **a** This figure illustrates the potential underlying mechanisms behind the associations between maternal medications and neonatal complications, utilizing significant adjusted odds ratios from PregMedNet and the PrimeKG Knowledge Graph, which contains scientifically validated biological interactions. Specifically, the subgraph containing three different node types—drugs, proteins/genes, and diseases—is shown here. Displayed is the subgraph of all nodes and edges that connect ondansetron to bronchopulmonary dysplasia via the shortest paths between the two nodes. This results in eight paths with twelve intermediary nodes. Each path represents a hypothesis for a possible underlying mechanism of action. **b** A particular path of interest suggests that ondansetron affects bronchopulmonary dysplasia through the protein interactions between CYP3A4 and IL-1${{{\rm{\beta }}}}$. This hypothesis builds on previous research showing the relationship between ondansetron and CYP3A, and a separate relationship between bronchopulmonary dysplasia and IL-1${{{\rm{\beta }}}}$. While there is limited evidence relating CYP3A4 to IL-1${{{\rm{\beta }}}}$ in the context of ondansetron and bronchopulmonary dysplasia, PregMedNet can generate hypotheses for such relationships by connecting established, but isolated biological knowledge using graph learning methods.

Notably, the mechanistic pathways underlying the association between maternal ondansetron exposure and neonatal BPD are highlighted here as a case study. PregMedNet generates plausible hypotheses involving interleukin-1${{{\rm{\beta }}}}$ (IL-1${{{\rm{\beta }}}}$), known as a key factor in BPD pathophysiology^41,42^. Hypotheses based on the protein/gene-only subgraph (Fig. 6) are particularly insightful, as they only include direct molecular interactions, avoiding non-specific biological entities (e.g., protein bindings), while the full biological interaction subgraphs (Supplementary Fig. 9) generate a broader range of hypotheses.

PregMedNet suggests that the shortest paths identified between ondansetron and BPD consist of 4-hop paths, meaning it takes four edges to reach the other side of the graph (Fig. 6a). A total of 8 shortest paths were identified, involving 12 intermediary nodes. Each path highlights potential mechanisms, visually represented by the edges between the nodes in the graph.

One of these paths suggests that ondansetron and BPD are connected via an interaction between IL-1${{{\rm{\beta }}}}$ and Cytochrome P450 3A4 (Fig. 6b). While there are prior research studies linking IL-1${{{\rm{\beta }}}}$ to arrested lung development in both human and experimental BPD^41,42^, ondansetron to multiple forms of cytochrome P450 (CYP450)^43,44^, and CYP450 to IL-1${{{\rm{\beta }}}}$^45^, the mechanism by which maternal ondansetron might impact neonatal BPD risk remains to be fully elucidated. As such, PregMedNet, in this instance, generates testable hypotheses by connecting proven yet isolated biological knowledge using graph learning methods. This approach prioritizes 8 specific hypotheses from countless possibilities, providing a targeted framework for subsequent experimental validation.

While we highlighted one of the potential MoA here, all 153 associations and their potential mechanisms are available on our interactive website.

## Discussion

PregMedNet provides a multifaceted perspective on maternal medication safety and neonatal complications in previously underrepresented populations—pregnant women and neonates. Its contributions include: (a) systematically analyzing maternal medication associations with neonatal complications, (b) adjusting for confounding variables across multiple medication-disease pairs, (c) providing in vivo experimental support for one of the generated hypotheses, and (d) suggesting potential mechanisms of action (MoA).

The systematic analysis of multiple maternal medications and neonatal complications was conducted by developing mother-baby dyads using retrospective claims data. We advanced our previous algorithms^17^ for linking mother and baby records, estimating gestational age and preterm birth, and incorporating multiple disease features based on International Classification of Diseases (ICD) and Current Procedural Terminology (CPT) codes. While there are previous perinatal drug safety studies using claims databases^46^, most have focused on short-term outcomes like live birth and stillbirth^17,19,20,47,48^ or examined a few maternal medication and neonatal complication pairs using traditional statistical testing^26^. In contrast, we systematically analyzed the impact of all noted maternal medications on 24 different neonatal complications in the cohort, which not only yielded an extensive set of results but also enabled us to characterize patterns of intricate associations across numerous drug-disease pairs. Additionally, PregMedNet further investigates maternal medication effects at finer resolution by retrieving drug information in a data-driven manner at the compound level, and by distinguishing between drug routes of administration. This approach differentiates distinct use cases for the same medication, such as topical betamethasone for skin inflammation and intramuscular betamethasone for preventing complications in preterm labor.

Another key contribution of the study is the adoption of a machine-learning-based confounding adjustment framework, which systematically selects and adjusts confounding variables for each of the 27,648 maternal medication-neonatal complication pairs. This approach aimed to produce more robust associations while minimizing reliance on manual selection, making it well-suited for large-scale systematic analyses. The study captured both established and additional potential associations. Notably, the framework aligned with previously reported clinical findings, such as the increased risk of neonatal respiratory complications in babies perinatally exposed to SSRIs^27^ and the increased risk of NAS in babies exposed to opioids^49^. These results illustrate the utility of the confounding adjustment framework in handling complex data. This framework is expected to scale to a wider range of systematic research studies, including those leveraging claims data or EHR.

Furthermore, the study examined potential additive effects of concurrent use of maternal medications on neonatal complications through retrospective DDIs analysis. Notably, the analysis indicated that certain known medication pairs (acetaminophen/oxycodone, NPH/short-acting insulins) were associated with a lower observed frequency of neonatal complications compared to their individual use. While there have been previous DDI research studies utilizing EHR, these studies have typically concentrated on already known side effects^50,51^, being based on data from a single institution^50,52^, or mainly focused on a limited number of medication pairs^51,53,54^. In contrast, we agnostically tested the effects of drug combinations without relying on prior knowledge, using a large-scale claims database from multiple institutions. This approach facilitates the discovery of interaction patterns and supports more unbiased analyses, which is particularly important given the increasing prevalence of polypharmacy among pregnant women over the past three decades^1^.

Notably, one of the findings uncovered by PregMedNet—the potential association between maternal ondansetron and neonatal BPD—was further explored through laboratory experimentation. Our preliminary in vivo experiments in mice observed that maternal ondansetron administration was associated with alterations in neonatal lung development. Specifically, increased MLI was noted, which could reflect defects in postnatal alveolar septation. Importantly, maternal ondansetron did not appear to affect neonatal survival or weight gain—factors that could otherwise exert secondary influences on lung development—suggesting that the observed effects.

Ondansetron is a potent and selective antagonist of the 5-HT3A serotonin receptor^55^. In the developing human lung, the 5-HT3A receptor is expressed on epithelial, endothelial, and smooth muscle cells throughout the canalicular, saccular, and alveolar stages of lung development^56^. However, the role of serotonin signaling through the 5-HT3A receptor in guiding normal lung morphogenesis is not yet understood. The increased MLI observed in ondansetron-treated mice, as predicted by the PregMedNet findings, suggests that the 5-HT3A receptor may play a role in normal lung morphogenesis.

As such, this in vivo experiment serves as a proof-of-concept, demonstrating PregMedNet’s capacity to generate biologically plausible hypotheses from large-scale claims data. While previous research studies have utilized claims databases to investigate maternal medication safety^17,19,20,26,46–48^, this study extends prior work by experimentally evaluating and providing preliminary support for claim-based hypothesis generation in perinatal medication safety. Notably, this experimentation also generated follow-up hypotheses regarding the previously unknown role of the 5-HT3A receptor in lung development. This further highlights PregMedNet’s potential to guide subsequent hypothesis-driven research. Further investigations into other PregMedNet-generated hypotheses will elucidate mechanisms and identify potential alternatives or treatments.

Understanding a drug’s MoA is critical for developing new treatments and preventing side effects^57,58^. However, traditional approaches to uncovering MoAs are complex and resource-intensive, often spanning years, and requiring in-depth hypotheses for testing^58^. PregMedNet generates hypotheses of MoAs for associations identified in this study that lack prior experimental validation by integrating real-world associations and a knowledge graph. A knowledge graph aggregates a wide range of experimentally supported molecular and biological interactions into a unified network, enabling a holistic view of biological relationships at a systems level. For significant medication-outcome associations lacking direct known interactions, we assume potential biological connectivity through intermediate entities and apply shortest-path analysis to identify the most direct biological routes. Grounded in the principle of parsimony^59^, this approach prioritizes shortest paths as the most biologically plausible connections. While exploratory, these shortest paths serve as a hypothesis-prioritization tool, highlighting a minimal set of candidate nodes for downstream experimental analysis. A case study with ondansetron and BPD showcased the plausibility of the hypothesis. Although there are previous research studies using graph learning methods to identify drug treatment mechanisms^60^ and polypharmacy drug side effects^60^, these efforts have relied solely on biological interactions from publicly available databases. PregMedNet distinguishes itself by connecting associations based on a large, real-world patient dataset to a knowledge graph to identify potential MoA. PregMedNet’s ability to suggest potential MoAs is expected to facilitate more efficient and targeted scientific investigation into underlying biological mechanisms.

Our study was subject to several limitations inherent to the database, the Merative™ Marketscan® Commercial Database. These include the restricted inclusion of patients with private insurance, excluding those without coverage, and the focus on prescribed outpatient medications, which omits inpatient and over-the-counter medications. In addition, critical demographic and behavioral variables—such as race, smoking habits, alcohol use, weight, and height are unavailable in claims data. The list of unavailable variables and their potential impact on association estimates is summarized in Supplementary Data 3. Moreover, there are also limitations inherent to claims data, including challenges in excluding coding errors introduced during billing processes and difficulties in confirming patient compliance with prescribed medication regimens. The limited specificity of ICD and CPT codes presents further challenges, as rare diseases often lack dedicated codes and are grouped under broader categories. For example, in ICD-9, BPD is categorized under code 770.7, described as “Chronic Respiratory Disease arising in the perinatal period,” while in ICD-10, it has a more specific code, P27.1, described as “Bronchopulmonary Dysplasia originating in the perinatal period”. To minimize coding challenges and improve accuracy, three clinicians reviewed the final list of codes after extracting the ICD and CPT codes.

The cohort definition adopted in this study entails several limitations. We restricted the cohort to singleton, first pregnancy live births to better isolate the impact of maternal medication on neonatal outcomes, while minimizing potential confounding from multiple gestations, multiparity, and spontaneous abortion. Multiple gestations and multiparity introduce shared intrauterine factors and intra-maternal correlations that are difficult to fully disentangle. Furthermore, including spontaneous abortions would introduce significant genetic confounding, as approximately half of early pregnancy loss is due to chromosomal abnormalities^61^ —factors that are frequently underreported and challenging to classify in clinical datasets. While these exclusion criteria ensure high internal validity for our primary analysis, they may limit the generalizability of the findings to more complex pregnancies. Therefore, the impact of maternal medication in these specific subgroups—such as multiple gestations or cases involving early pregnancy loss—remains an important area for further investigation. Additionally, the potential influence of paternal comorbidities and medication exposure on neonatal complications should be considered, as emerging evidence suggests that paternal health conditions and pharmacologic exposures may influence offspring outcomes^62,63^. Future research incorporating more granular, linked clinical data—including medication dosing information and treatment duration—is warranted to establish a truly comprehensive reproductive safety profile for these medications.

An additional limitation to consider is the complexity and variability of the confounding structure for each association, which may not be fully addressed in the systematic analysis provided by PregMedNet. This may lead to the inclusion of non-causal associations, such as confounding by the indication for the medication and its severity, both of which can vary between associations.

As such, the study has inherent limitations, despite our efforts to maximize the utility of the available data to provide the best possible estimates. To fully establish causal relationships, follow-up analyses aimed at causal inference that incorporate more accurate data along with detailed confounding factors for each association will be necessary. Nonetheless, PregMedNet significantly narrowed the scope to 261 associations involving 68 maternal medications from an initial 27,648 associations, enabling prioritization for further causal investigation. Importantly, our experimental investigation of one of the associations identified in this study supports the plausibility of our findings. Taken together, these findings demonstrate PregMedNet’s value as a platform for robust hypothesis generation despite these limitations.

It is also important to consider that medications with statistically non-significant associations in our dataset are not necessarily safe; limited usage of medications already known to pose risks among expectant mothers may reduce the statistical power to assess these associations. We analyzed the frequency of medication use across Pregnancy Categories based on Australian categories for prescribing medicine in pregnancy^64^ (see Supplementary Data 4), which shows that prescription frequency decreases as the risk category increases from A to X. Category X medications (high risk of causing permanent damage to the fetus) were prescribed to only 36 patients, whereas category A medications (drugs without any proven damage to the fetus) were prescribed to 213,019 patients. This distribution supports the notion that higher-risk medications are prescribed less frequently, resulting in less statistical power and explaining why they may not appear in the results. This trend is also reflected in the DDI results, where fewer statistically significant DDIs are observed due to small cohort sizes per drug combination. Thus, non-significant DDIs do not necessarily indicate a lack of effect. However, significant findings in PregMedNet provide risk estimates for commonly used maternal medications, which can further guide decisions on perinatal medication safety.

To further contextualize these power-related limitations, we report that significant associations were identified with a minimum dyad count of 11 exposure cases for odds ratios (ORs, aORs) and 12 for drug–drug–disease interactions (DDIs). This is consistent with the CMS Limited Data Set (LDS) Data Use Agreement (DUA)^65^, which restricts the reporting of results derived from cohorts with fewer than 11 cases to protect patient privacy. This indicates that while PregMedNet is capable of identifying risks even within small cohorts near the reporting threshold, associations involving rarely prescribed medications or uncommon drug combinations may fall below this limit, remaining undetectable or unreported due to statistical and privacy constraints. Among the significant findings, the median number of exposure cases was 81 (IQR: 30–277.75) for raw ORs, 656 (IQR: 325–1545) for adjusted ORs, and 35 (IQR: 34–45) for DDIs. The distribution of the number of exposure cases for ORs and aORs is depicted in Supplementary Fig. 10. The shift of the median exposure count from 81 to 656 after adjustment suggests that only associations with a larger number of exposures retained statistical significance after controlling for confounders, as the adjustment process inherently requires more robust evidence to overcome potential bias. Taken together, while acknowledging these inherent constraints regarding rarely used medications, our findings provide a robust overview of perinatal medication risks, with reported associations interpreted within the context of exposure prevalence, statistical power, and reporting thresholds.

Finally, while the knowledge graph used for MoA discovery aggregates comprehensive and verified biological associations, it currently exists in a binary setting, lacking granular information regarding the relative strength, directionality, and cell-specific context of these interactions. In this context, applying highly expressive graph machine learning may yield representations driven by network topology—such as node centrality or hubness—rather than real-world biological strength, which could introduce unintended artifacts. Therefore, we utilized a transparent, shortest-path-based approach for MoA hypothesis generation to maintain parsimony and interpretability. Future extensions of PregMedNet incorporating granular metadata, coupled with the application of advanced graph-based machine learning, will further enhance the predictive power and precision of MoA-based hypothesis generation.

PregMedNet provides a multifaceted approach to studying the impact of perinatal drug use on neonatal outcomes, an area previously underexplored. Using large, multi-institutional claims data and the systematic approach developed in this study, we identified robust associations, one of which was supported by in vivo experimentation. PregMedNet also generated hypotheses on potential MoAs for the associations. The extensive results of PregMedNet are available through an interactive website (https://pregmednet.stanford.edu/). We expect PregMedNet to contribute to advancing maternal and neonatal healthcare by providing insights that guide safer medication use during pregnancy and help reduce the risk of neonatal complications.

## Methods

This research complies with all relevant ethical regulations. This study was reviewed and approved by the institutional review board (IRB) at Stanford University (no. 39225). The requirement for informed consent was waived because the study used de-identified, retrospective data and posed minimal risk to participants, in accordance with applicable regulations. No participant compensation was provided. All animal experiments were approved by Stanford University’s Institutional Animal Care and Use Committee (IACUC) under protocol no. 33897, and were conducted in accordance with relevant institutional and national guidelines and regulations.

### PregMedNet

PregMedNet is a platform designed to assess the impact of maternal medication use during pregnancy on neonatal complications. PregMedNet provides a comprehensive analysis of the multifaceted associations between 1152 maternal medications used during pregnancy and 24 neonatal complications. These associations were systematically examined through retrospective analysis of large-scale claims data using the advanced machine learning framework. Notably, one of the identified associations was further examined in vivo, providing experimental support for the findings. Additionally, possible underlying mechanisms of discovered correlations between diseases and medications were identified using the Knowledge Graph and graph learning methods. The entire results are open source and accessible through an interactive website (https://pregmednet.stanford.edu/). The overall analytical pipeline is summarized in Box 1.

Box 1 PregMedNet analytical pipeline **1. Cohort construction:** Construct mother–newborn dyads from a de-identified, multi-institutional claims database.

**2. Feature extraction:** Extract maternal medication exposures, maternal comorbidities, neonatal complications, and six additional covariates (maternal age, gestational age, preterm birth, infant sex, healthcare facility region, and healthcare facility geographic location).

**3. Unadjusted association analysis:** Systematically estimate associations between maternal medication exposures and neonatal complications using **odds ratios (ORs)**.

**4. Adjusted association analysis:**

**a.** **Confounder selection:** Address multicollinearity using **variance inflation factor (VIF)**-based selection of independent variables.

**b.** **Feature engineering:** Normalize maternal/gestational age via **min–max scaling** and transform healthcare facility geographic location using **binary encoding**.

**c.** **Outcome-specific adjustment:** Additionally, exclude gestational age and preterm birth from the confounder set when modeling postmaturity to prevent multicollinearity.

**d.** **Adjusted modeling:** Systematically estimate adjusted odds ratios (**aORs**) using **LASSONoExp**—a modified LASSO framework where the exposure variable’s coefficient remains unpenalized.

**5. Drug–drug interaction (DDI) analysis:** Systematically evaluate interaction effects between co-administered maternal medications using logistic regression-based methods.

**6. Replication and experimental support:**

**a.** **Computational replication:** Replicate steps 3–5 in an independent validation cohort; assess results via **Pearson correlation**.

**b.** **In vivo experiment:** Experimentally support a key association identified in the computational analyses—between maternal ondansetron use and neonatal bronchopulmonary dysplasia (BPD)—using a mouse model.

**7. Mechanism inference:** Utilize **knowledge graphs** and **graph-based learning** to explore potential biological mechanisms underlying the identified associations.

**8. Data dissemination:** All findings and inferred mechanisms are integrated into an interactive web platform (https://pregmednet.stanford.edu/).

### Data preparation

Mother and baby cohorts, along with their medical condition and medication histories, were extracted from the large-scale, de-identified claims data of the Merative™ Marketscan® Commercial Database^21^. This database houses de-identified medical, medication, and dental claim records for over 250 million patients in the US. We accessed the data through the Stanford Center for Population Health Sciences. A conceptual flow diagram of the data preparation process is shown in Supplementary Fig. 1 with details in Supplementary Fig. 2.

#### Selection of the mother and baby cohorts

We identified a cohort of women aged 12–55 years with singleton pregnancies using the International Classification of Diseases, Ninth Revision (ICD-9), ICD-10, and diagnosis-related group (DRG) codes from 2007 to 2021 (Supplementary Data 5). We included only the first pregnancies to avoid dependencies among women with multiple pregnancies. Molar and ectopic pregnancies, as well as spontaneous and induced abortions, were excluded (Supplementary Data 6). Only mothers continuously enrolled in the insurance plan for 90 days prior to delivery were included to ensure all maternal records are captured without any omissions.

The newborn cohort was generated based on the newborn codes from ICD-9 and ICD-10 from 2007 to 2021 (Supplementary Data 7). Only babies continuously enrolled in the insurance plan for 90 days after delivery were included to ensure newborn records are captured without any omissions.

#### Extraction of medical conditions and medications

For each mother, all medical claims records for 90 days prior to delivery were extracted from both the inpatient and outpatient claims tables. From these records, we identified 68 maternal conditions of interest (Supplementary Data 8), defined using ICD codes extracted through either syntax-based keyword searches or manual selection, when required, from publicly available code lists^66–68^. In addition, we obtained maternal age, gestational age at delivery, preterm birth, and the region and geographic location of the healthcare facility. All maternal medication records during the same period were extracted from the prescription drug claims table as National Drug Codes (NDCs). These medications were further processed as described in the “Maternal medications processing” section.

For each newborn, medical claims records for 90 days after delivery were extracted. ICD codes from publicly available lists^67,68^ were used to define 24 neonatal complications (Supplementary Data 9), which were reviewed by three clinicians. Neonatal sex of each baby, assigned at birth, was also extracted. Maternal data and corresponding baby data were linked using a Family ID, indicating which baby was delivered by which mother (Fig. 1a).

#### Estimation of gestational age and preterm birth

Gestational Age (GA) at delivery was determined using GA codes, which are ICD codes that represent the number of completed weeks of gestation (Supplementary Data 10). ICD codes indicating preterm birth (PTB) were designated as PTB codes (Supplementary Data 11). We searched maternal records for both GA and PTB codes spanning one month before to one month after the delivery date. This wide temporal window was necessary to account for the occasionally inaccurate timing of claims data.

GA estimation proceeded as follows:

1. If a single GA code was recorded in maternal records, that value was extracted as the GA. If multiple GA values are recorded, the highest recorded GA value is selected.
2. If no GA code was recorded in maternal records, but infants had a GA code in their records within one month of delivery, GA was inferred from the infant record.
3. If no GA code was recorded in both maternal and infant records, but PTB codes were recorded, a GA of 34 weeks was assigned. Otherwise, a GA of 39 weeks was designated.

Preterm birth records were estimated similarly:

1. If PTB codes were recorded in maternal records, the pregnancy was associated with PTB.
2. If no PTB codes were recorded in maternal records, but the GA code suggested less than 37 weeks of gestation, the pregnancy was considered to be delivered preterm. Otherwise, a full-term delivery was assumed.
3. If mothers had both PTB codes and GA codes, but GA was higher than 37 weeks, they were removed from the cohort.

#### Maternal medications processing

Generic drug names, drug classes, and route of administration information from MarketScan Redbook^69^ were additionally integrated into the maternal medication records using NDC. Medication records were further processed as follows (Supplementary Fig. 3):

1. Categorized medication claims records by their route of administration, identifying 24 distinct routes.
2. For each route of administration, generic medication records were split at the compound level using syntax-based parsing of slashes and semicolons.
3. Next, medication names were standardized using DrugBank IDs^70^ to account for compounds described differently (e.g., Oxycodone Hydrochloride vs. Oxycodone HCl). Medications without DrugBank IDs, totaling 139, were manually reconciled. Out of these, 31 medications were assigned manual IDs, while the rest were discarded.
4. Each final medication consisted of a harmonized medication name and route of administration such that the same medication taken by different routes of administration was considered separately. Medication administration was binarized so that each medication was designated as taken or not taken.

#### Final mother-baby cohort

The final dataset included 1152 maternal medications and 68 maternal covariates for 90 days prior to delivery, as well as 24 neonatal complications for 90 days after delivery. It also included 6 additional attributes: maternal age, gestational age at delivery, preterm birth, baby’s sex, and both the region and geographic location of the healthcare facility. Except for maternal age, gestational age at delivery, region, and geographic location, all other variables in the dataset were binary variables, indicating the presence or absence of a condition or medication.

The final dataset was split into a discovery cohort and a validation cohort. Babies delivered between 2007 and 2015 were included in the discovery cohort, while those delivered between 2016 and 2021 were used for validation.

### Odds ratios: maternal medications and neonatal complications

The associations between maternal medications during pregnancy and neonatal complications were calculated using both unadjusted and adjusted odds ratios. To comply with the CMS Limited Data Set (LDS) Data Use Agreement (DUA)^65^ to protect patient privacy, the calculation was conducted only when the number of exposure cases (i.e., the count of patients with both the medication exposure and the specific neonatal outcome) was more than 10. Odds ratios were derived from logistic regression, while adjusted odds ratios were determined through Lasso Logistic Regression Without Penalizing Exposure Variable (LassoNoExp)^71,72^. Potential confounders were selected using variance inflation factors (VIF) from among 68 maternal comorbidities and 6 additional attributes (maternal age, gestational age at delivery, preterm birth, and the region and geographic location, the sex of each baby) to mitigate the effects of multicollinearity. We next describe this approach in detail.

#### Unadjusted odds ratios

Unadjusted Odds ratios (ORs) were computed using univariate logistic regression for each medication-disease pair. In Eq. (1), ${{{\bf{Y}}}}\in \{0,1\}^{817,402}$ represented the neonatal complication vector, and ${{{\bf{M}}}}\in \{0,1\}^{817,402}$ denoted the medication vector where entry ${M}_{j}$ equaled 1 if the mother indexed by j was exposed to the medication, and 0 otherwise. Therefore, the odds ratio for each pair was given by Eq. (2). This process was repeated for all possible pairs, yielding 27,648 ORs from 1152 medications and 24 diseases. The Benjamini-Hochberg procedure^73^ was subsequently applied to control the false discovery rate (FDR) at 5% to correct for multiple comparisons.

- Logistic Regressions between each medication and neonatal complication pair
  1 $$\log [\frac{{{{\rm{P}}}}({{{\bf{Y}}}}=1|{{{\bf{M}}}})}{{{{\rm{P}}}}({{{\bf{Y}}}}=0|{{{\bf{M}}}})}]=\,\log ({{{\rm{odds}}}})={\beta }_{0}+{\beta }_{1}{{{\bf{M}}}}$$
- Odds Ratio

2 $${{{\rm{OR}}}}=\exp ({\beta }_{1})$$

#### Adjusted odds ratios

While ORs provided an estimate of the effects of maternal medication, they could be influenced by spurious associations due to unaccounted confounders. However, traditional methods of manually selecting confounders were impractical for our study of 27,648 associations, and uniformly adjusting for high-dimensional confounders also posed a challenge due to overadjustment bias^74^ and multicollinearity^75^. To obtain a more accurate representation of the maternal medication’s impact on neonatal complications, we adjusted the odds ratios for 74 selected potential confounders. These adjusted odds ratios (aORs) were computed using Lasso Logistic Regression Without Penalizing Exposure Variable (LassoNoExp) after removing certain covariates to mitigate multicollinearity.

##### Step 1: Managing multicollinearity of confounders

Multicollinearity refers to correlation of independent variables in multiple regression analysis. This poses challenges for both mathematical modeling and results interpretation. For a given multiple regression ${{{\bf{y}}}}={{{\bf{X}}}}{{{\boldsymbol{\beta }}}}+{{{\boldsymbol{\epsilon }}}}$, the ordinary least squares (OLS) is given by $\hat{{{{\boldsymbol{\beta }}}}}={({{{{\bf{X}}}}}^{{{{\rm{T}}}}}{{{\bf{X}}}})}^{-1}{{{{\bf{X}}}}}^{{{{\rm{T}}}}}{{{\bf{y}}}}$. However, if columns of ${{{\bf{X}}}}$ are linearly dependent and therefore correlated, then ${{{{\bf{X}}}}}^{{{{\rm{T}}}}}{{{\bf{X}}}}$ becomes non-invertible (singular), making it impossible to complete the OLS formula. Highly correlated columns are also difficult to interpret because it becomes difficult to discern the individual contributions of each factor to predicting the dependent variable, and increases the variance of the regression coefficient, causing it to become unstable^76^.

In our study, we initially included a total of *k* = 74 potential confounders, encompassing maternal comorbidities and demographic variables, identified based on previous research related to maternal morbidity^77^. To reduce multicollinearity, we first selected independent variables (i.e. confounders) based on the Variance Inflation Factor (VIF)^78^. VIF is a measure of multicollinearity that indicates the degree of intercorrelation among the independent variables. VIF of each confounder was calculated by fitting a linear regression model using one confounder as the dependent variable and all others as independent variables (3). Subsequently, the multiple correlation coefficient of ${i}^{{th}}$ confounder ${{{{\bf{X}}}}}_{i}$, ${{R}_{i}}^{2},$ was used to calculate VIF of ${i}^{{th}}$ confounder ${{{{\bf{X}}}}}_{i}$, as given by (4). This process was repeated for all confounders, with a higher VIF indicating greater multicollinearity.

3 $${{{{\bf{X}}}}}_{i}={\beta }_{0}+{\beta }_{1}{{{{\bf{X}}}}}_{1}+\cdot \cdot \cdot+{\beta }_{k}{{{{\bf{X}}}}}_{k}$$

4 $${{{\rm{VI}}}}{{{{\rm{F}}}}}_{i}=\frac{1}{1-{{R}_{i}}^{2}}$$

In our model, we excluded six confounders that had either a VIF greater than 5 or zero vectors^79^. This exclusion included specific substance use disorders (e.g., fentanyl, amphetamine, ecstasy, methamphetamine, methylphenidate) and region of the healthcare facility features. Nonetheless, we retained both gestational age and maternal age, despite their VIFs exceeding 5, due to their critical role in determining outcomes. After this process, a total of 68 confounders remained, which were used for adjustment.

##### Step 2: Feature engineering: demographic features

Maternal age, gestational age, and the geographic location of the healthcare facility features were further processed. Because maternal age and gestational age had a wider span of values compared to binary inputs, which could have led to them being overemphasized in the model, they were scaled using a min-max scaler. The geographic location of the healthcare facility was a categorical variable with 54 categories, which can introduce additional multicollinearity. Thus, it was encoded using a binary encoding method, expressing 54 variables in 6 columns. The final dataset for adjusted odds ratios contained 73 columns representing confounders, along with 1152 maternal medications and 24 neonatal complications.

##### Step 3: Additional adjustment for postmaturity

When the outcome variable y was postmaturity, preterm birth and gestational age were excluded from the confounders due to their potential to introduce multicollinearity, as both were defined by gestational age. As a result, the total number of confounders for postmaturity was reduced to 66, compared to 68 for the other 23 neonatal complications. This adjustment led to a final dataset containing 71 columns representing confounders, along with 1152 maternal medications, specific to postmaturity, distinguishing it from the datasets used for the other neonatal complications.

##### Step 4: LASSONoExp: adjusted odds ratios calculation

We adjusted the confounders using LASSONoExp, an algorithm based on LASSO regression^80,81^. Lasso regression is effective for adjusting potential confounders and estimating treatment effects due to its ability to select variables among high-dimensional confounders and reduce overfitting^82^. LASSONoExp is the same as the LASSO, except that the regression coefficient of the exposure variable, in our case, maternal medication exposure, is not penalized in the model. This prevents the coefficient of maternal medication from yielding a zero value. Consequently, the exposure effect of the estimator is not biased, while the effects of the confounding variables are regularized^71,72^.

Let ${{{\bf{M}}}}$ denote the maternal medication of interest, as defined in Eq. (1). As before, ${{{\bf{Y}}}}$ represents the neonatal complications of interest, and ${{{{\bf{X}}}}}_{i}\,$(where $2\le i\le P$) indicates the confounders that were selected from Step 1 to Step 3 (in our case, P=71 for postmaturity, and P=73 for the rest of the neonatal complications) such that ${X}_{{ij}}=1$ if the mother indexed by j had comorbidity i and 0 otherwise. The model is then given by

5 $${{{\bf{Y}}}}={\beta }_{0}\bullet 1+{\beta }_{1}{{{\bf{M}}}}+{\sum }_{i=2}^{P}{\beta }_{i}{{{{\bf{X}}}}}_{{{{\bf{i}}}}}$$

Coefficients ${\beta }_{i}$ are determined by

6 $${{{\rm{argmi}}}}{{{{\rm{n}}}}}_{\beta }\left({{{{\rm{||}}}}{{{\bf{y}}}}-{{{\bf{Y}}}}{{{\rm{||}}}}}_{2}^{2}+\lambda {\sum }_{j=2}^{P}\left|{\beta }_{j}\right|\right)$$

7 $${{{\rm{O}}}}{{{{\rm{R}}}}}_{{{{\rm{adjusted}}}}}=\exp ({\beta }_{1})$$

In LASSONoExp, the coefficients ${\beta }_{i}$, as denoted in (5), are obtained by solving the objective function (6), where hyperparameter λ represents a penalty weight for the confounders. In (6), the term ${{{\bf{y}}}}$ represents the ground truth (actual observed values), whereas ${{{\bf{Y}}}}$ signifies the predicted values obtained from the model. Here, ${\beta }_{1}$, the coefficient of maternal medication, is not penalized, while ${\beta }_{2}$ to ${\beta }_{P}$ are penalized based on the penalty weight. Adjusted odds ratios are then given by (7).

We repeated this process for the 1446 maternal medication and neonatal complication pairs that yielded statistically significant unadjusted odds ratios (ORs). The Python package statsmodels was used to fit the model. The penalty weight, λ, was selected for each maternal medication-neonatal complication pair based on 3-fold stratified cross-validation. This selection involved ten coefficients ranging between 0.01 and 10, using the Python package optuna. Finally, the Benjamini-Hochberg procedure was applied to control the FDR at 5%, correcting for multiple hypothesis testing.

#### Network graph

Unadjusted odds ratios (ORs) and adjusted odds ratios (aORs), given by Eqs. (2) and (7) respectively, were visualized as a network graph. This visualization was chosen to illustrate the pattern of interactions and to allow exploration of multiple correlations in a single plot, given that there are more than 1000 statistically significant correlations (see Fig. 2, Supplementary Fig. 4, and interactive graphs at http://pregmednet.stanford.edu). In the figures, each node represents either neonatal complications or maternal medications, while the edges between them indicate statistically significant odds ratios. The size of the nodes is proportional to the number of edges connected to them, and the width of the edges is inversely proportional to the *p*-values of the odds ratios. The color of the nodes represents either the medication group or disease (with gray-colored nodes at the center), and the color of the edges indicates whether the odds ratios are positive (gray) or negative (blue). The locations of the nodes were estimated based on t-distributed Stochastic Neighbor Embeddings (t-SNE)^83^ within each disease or medication group, demonstrating relative similarities in the patterns of medication use or disease outbreaks in mother-baby dyads. The Python packages scikit-learn, network, and bokeh were used to calculate t-SNE, construct network graphs, and generate interactive graphs, respectively.

#### Verification of results in the validation cohort

To ensure the reproducibility of the odds ratios and adjusted odds ratios, we repeated the same process on the validation cohort and compared the results of unadjusted and adjusted odds ratios between the two cohorts by matching maternal medications and neonatal complications. After matching, scatter plots were created, and Pearson correlation coefficients were calculated between discovery and validation cohort odds ratios.

### Drug–drug interactions (DDIs)

#### Derivation of DDIs

Drug–drug interactions (DDIs) occur when two or more medications react with each other, affecting the outcome in a way that would otherwise not occur. DDIs are considered in pairs of maternal medications relative to each of the 24 neonatal complications. This is done to determine whether interactions between two medications could additionally affect neonatal complications. Gestational Age, one of the strongest confounders, is included and adjusted for in the analysis. DDI effects were captured using a logistic regression-based model^84^, given by:

8 $${{{\bf{Y}}}}={\beta }_{0}+{\beta }_{1}{{{{\bf{M}}}}}_{1}+{\beta }_{2}{{{{\bf{M}}}}}_{2}+{\beta }_{3}{{{{\bf{M}}}}}_{1}{{{{\bf{M}}}}}_{2}+{\beta }_{4}{{{\bf{GA}}}}$$

where ${{{\bf{GA}}}}$ denotes gestational age, ${{{{\bf{M}}}}}_{i}(i={{\mathrm{1,2}}})$ denotes exposure to maternal medication i, and ${{{\bf{Y}}}}$ represents the neonatal complication of interest. ${{{{\bf{M}}}}}_{1}{{{{\bf{M}}}}}_{2}$ represents the interaction between medication 1 and medication 2, and its coefficient, ${\beta }_{3}$, indicates the direction and magnitude of this interaction. If ${\beta }_{3}$ is less than 0, it suggests that the interaction tends to reduce the risk of neonatal complications. Conversely, a ${\beta }_{3}$ greater than 0 indicates that the interaction increases the risk of neonatal complications. The higher absolute value of ${\beta }_{3}$ suggests stronger interactions.

We repeated this process for all maternal medication pairs among 1152 medications for 24 neonatal complications. We only considered interactions where there were more than 10 mother-baby dyads where the mothers took both medications, and the babies had neonatal complications. A total of 28,417 maternal medication pairs and neonatal complications out of the 15,911,424 all possible pairs (calculated as $\left({0ex}{1152}{2}\right)\times 24$) were analyzed. The Benjamini-Hochberg procedure was then applied to control the FDR at 5%, correcting for multiple hypothesis testing. Ultimately, a total of 121 maternal medication pairs and neonatal complications showed statistically significant ${\beta }_{3}$.

#### Calculate the odds of drug pairs

For the 121 maternal medication pairs and neonatal complications that exhibited statistically significant ${\beta }_{3}$ values, odds ratios for concomitant medication use were calculated as:

9 $${{{\rm{O}}}}{{{{\rm{R}}}}}_{1}=\exp ({\beta }_{1})$$

10 $${{{\rm{O}}}}{{{{\rm{R}}}}}_{2}=\exp ({\beta }_{2})$$

11 $${{{\rm{O}}}}{{{{\rm{R}}}}}_{12}=\exp ({\beta }_{3})\times {{{\rm{O}}}}{{{{\rm{R}}}}}_{1}\times {{{\rm{O}}}}{{{{\rm{R}}}}}_{2}$$

As with ${\beta }_{3}$, the Benjamini-Hochberg correction was subsequently applied to ${\beta }_{1}$ and ${\beta }_{2}$, respectively, to control the FDR at 5%. Equation (11) was applied when all ${\beta }_{3}$, ${{{{\rm{OR}}}}}_{1}$, ${{{{\rm{OR}}}}}_{2}$ are statistically significant. Finally, we extracted the medication pairs that showed statistically significant ${{{\rm{O}}}}{{{{\rm{R}}}}}_{12}$ among the 121 medication pairs, and then compared these results with the odds ratios of their corresponding single medication use cases.

#### Final DDIs overlap: discovery vs. validation cohorts

The same process was repeated within the validation cohort to identify significant values of ${\beta }_{3}$ and ${{{\rm{O}}}}{{{{\rm{R}}}}}_{12}$. DDIs for which both ${\beta }_{3}$ and ${{{\rm{O}}}}{{{{\rm{R}}}}}_{12}$ were found to be significant in both the discovery and validation cohorts were extracted for further analysis.

### In vivo ondansetron experiments

The association between maternal ondansetron and neonatal bronchopulmonary dysplasia, which was significant in both OR and aOR analyses, is tested experimentally.

#### Mice

Female and male C57BL/6J mice (Mus musculus; genetic background: C57BL/6) were purchased from The Jackson Laboratory and maintained in-house under standard housing conditions with a 12-h light/12-h dark cycle, ambient temperature of 18–23 °C, and humidity of 40%–60%. A total of 20 female and 10 male C57BL/6 J mice aged 8–12 weeks were used for timed matings. Neonatal pups aged P0–P11 were used for survival analysis and pup weight measurements, and pups at P11 were used for mean linear intercept analysis.

#### Ondansetron administration

Ondansetron (Millipore Sigma 1478571) was dissolved in PBS without calcium and magnesium (Gibco 10010-049). C57BL/6J female and male mice were timed mated, and pregnancy was confirmed by a 2 g increase in weight by E10^85^. Starting on E15, pregnant mice were dosed daily with either 5 mg/kg body weight of ondansetron or PBS by intraperitoneal (i.p.) injection until delivery of pups.

E15 was selected to align with the human ondansetron exposure window in PregMedNet, corresponding to the last 90 days of gestation in humans. This period overlaps with key stages of murine lung morphogenesis, providing a suitable window to investigate the potential linkage between ondansetron and abnormal lung development relevant to BPD.

Ondansetron can be administered to human patients via oral or systemic (intravenous) routes. For this study, we employed intraperitoneal injection to ensure precise dosing and bioavailability while minimizing handling stress for the pregnant dams.

The dose of 5 mg/kg body weight was determined based on clinical dosing guidelines for hyperemesis gravidarum (8 mg, 3-4 times daily)^23,86^. Human-to-mouse dose conversion was performed using guidelines taking into account differences in body surface area (BSA) and *K*_m_^87^. The 5 mg/kg dose in mice corresponds to an approximately 24 mg/day regimen for a 60 kg human. This dose also falls within the range (0.1–25 mg/kg) previously reported in murine studies^88–90^. To evaluate the impact on fetal lung morphogenesis, a 5–6 day dosing regimen was implemented. This duration captures critical developmental windows and remains within the scope of clinical guidelines for severe hyperemesis gravidarum, where extended treatment is clinically indicated^23^.

##### Lung collection for histology

On postnatal day P11, pups were euthanized and the lungs were inflated with 4% PFA (Electron Microscopy Sciences 15713). For comparing lung morphogenesis, pups were randomly selected from each litter, with each newborn mouse considered an individual experimental unit. Following inflation, the lungs were collected and fixed in 4% PFA for 1 h at room temperature. Quality control ensured that only lungs properly inflated during fixation without obvious, gross damage to lung tissue were processed and analyzed. The lungs were then transferred to 15% sucrose (Millipore Sigma S0389) and incubated overnight at 4 °C. The following day, the lungs were transferred to 30% sucrose and incubated for 3 days at 4 °C, followed by embedding in OCT mounting medium (Leica 14020108926) for cryo-sectioning. Lungs were sectioned at a thickness of 10 m.

##### Hematoxylin and eosin staining

For hematoxylin and eosin staining, slides were brought to room temperature and fixed in 10% buffered formalin (StatLab FXBFOLT) for 20 min. Slides were immersed in Gill II hematoxylin (Newcomer Supply 1180D) for 15 s, rinsed with water, and immersed in Scott’s bluing reagent (Ricca Chemical Company 66971) for 30 s. Slides were then rinsed with water, immersed in eosin Y (Millipore Sigma HT110116) for 2 min, and again rinsed with water. Slides were dehydrated with dips in ethanol (Fisher A962P-4) and cleared with dips in xylene (Fisher X5-4) before being mounted with non-aqueous mounting medium (Vector Laboratories H570060).

##### Mean linear intercept quantification

All sample processing, staining, imaging, and analysis were performed in a blinded manner. For quantification, brightfield images were captured with the Leica Thunder microscope using the 20X objective. Images were quantified on FIJI using the Mean Linear Intercept plug-in^91^. The image analysis parameter was set to 500 pixels between lines, and both horizontal and vertical chords were measured. All horizontal and vertical chords were averaged to get an image average value. For each pup, 3 representative lung sections were stained and imaged, with 5 images taken from each section for a total of 15 images quantified. Statistical analysis for Fig. 5e was performed using a *t*-test on all data points (135–180 images) to accurately assess the normal variation that occurs throughout regions of the lungs. As lung morphogenesis was analyzed in neonatal mice before sex could be accurately determined, sex was not considered prior to measurement, and groups likely included both male and female animals.

### Knowledge graph: hypothesis generation for new medication effects

The hypotheses of underlying mechanisms for medication effects identified in this study were explored using the Precision Medicine Knowledge Graph (PrimeKG)^40^. This publicly available knowledge graph integrates 20 different biological databases, providing a holistic view of the interactions among 10 different types of biological entities. In PrimeKG, biological entities are represented as nodes, and their interactions as edges. Using this framework, we tested two different types of subgraph configurations.

#### Subgraph configuration 1

First, we extracted a subgraph consisting of four biological node types: proteins/genes, biological processes, molecular functions, and cellular components. The subgraph included seven types of edges representing various interactions: protein–protein, bioprocess–protein, molfunc–protein, cellcomp–protein, bioprocess–bioprocess, molfunc–molfunc, and cellcomp–cellcomp.

Next, we incorporated the disease node of interest and its connections to proteins through disease-protein edges. When the disease node was unavailable, we replaced it with an effect/phenotype node.

To ensure the subgraph remained fully connected and did not contain isolated components, we iteratively expanded it by adding additional biological node types (phenotype, disease_phenotype_positive, disease_phenotype_negative) as needed. This expansion was performed per disease type, ensuring that all relevant nodes and edges formed a single, connected network.

We then incorporated the medication node of interest, linking it to proteins through drug-protein edges. Once the subgraph was fully constructed, we extracted all possible shortest paths between the two key nodes of interest—a maternal medication and a neonatal complication—using the Dijkstra algorithm^92^. The resulting list of shortest paths was considered indicative of potential underlying mechanisms, providing insights into how maternal medications might contribute to neonatal complications.

#### Subgraph configuration 2

For comparison, we conducted the same analysis using a simplified subgraph consisting of only three node types—drugs, proteins/genes, and diseases—and three edge types (drug-protein, protein-protein, and disease-protein). As before, we extracted all possible shortest paths between the maternal medication and the neonatal complication to infer potential mechanisms.

#### Iterative analysis and visualization

This process was repeated for all associations identified in this study between maternal medications and neonatal complications that exhibited statistically significant adjusted odds ratios. The resulting hypotheses were visualized as graphs using the Python packages networkx and bokeh.

### Statistics and reproducibility

Detailed descriptions of statistical analyses and the machine learning framework are provided in the relevant “Methods” sections, including “Odds ratios: maternal medications and neonatal complications” and “Drug–drug Interactions (DDIs)”. Information on data access, data preparation, and code availability is described in the “Data availability,” “Data preparation,” and “Code availability” sections, respectively, to ensure reproducibility.

No statistical method was used to predetermine sample size. No data were excluded from the analyses beyond predefined cohort criteria, such as the exclusion of non-singleton or ectopic pregnancies, as described in the “Data preparation” section. The study was based on retrospective observational data; therefore, experiments were not randomized, and investigators were not blinded to allocation during experiments and outcome assessment.

### Reporting summary

Further information on research design is available in the Nature Portfolio Reporting Summary linked to this article.

## Supplementary information

Supplementary Information

Description of Additional Supplementary Files

Supplementary Data

Reporting Summary

Transparent Peer Review file

## Data Availability

The data that support the findings of this study are not publicly available due to restrictions imposed by the data provider and the data use agreement. The dataset used in this study is the Merative™ Marketscan® Commercial Database, a proprietary dataset containing de-identified health information derived from private insurance claims. The data use agreement prohibits the redistribution of both raw and processed data. Access to the data is available to qualified entities (including academic, nonprofit, governmental, and commercial institutions) through purchase and a formal data use agreement with Merative™. Requests for access should be directed to the Merative™ Product Support Team (productsupport@merative.com). Additional information regarding data access and licensing is available at: https://www.merative.com/documents/brief/marketscan-explainer-general. The time required to obtain access may vary depending on the contracting process. The data are available for the duration of the licensing period. To facilitate reproducibility, detailed descriptions of data processing and analysis are provided in the “Methods” section, and the code used in this study is publicly available at: https://github.com/yerakim824/PregMedNet.

## Code availability

The PregMedNet framework and custom code used in this study can be accessed on GitHub (https://github.com/yerakim824/PregMedNet). A version of the code corresponding to this study has been archived on Zenodo (10.5281/zenodo.19228135)^93^. Analysis were performed in Python 3 (version 3.10) using key packages including scikit-learn (v1.2.2), statsmodels (v0.13.5), optuna (v3.4.0), bokeh (v2.4.3), and NetworkX (v3.2.1).
