# Supplementary material for: PregMedNet: Multifaceted maternal medication impacts on neonatal complications
Source: Nat Commun. 2026 Jul 10;17:8526. doi: 10.1038/s41467-026-75000-0 (PMC13483950; doi:10.1038/s41467-026-75000-0)
Supplement: Supplementary file 1 [file 41467_2026_75000_MOESM1_ESM.pdf]

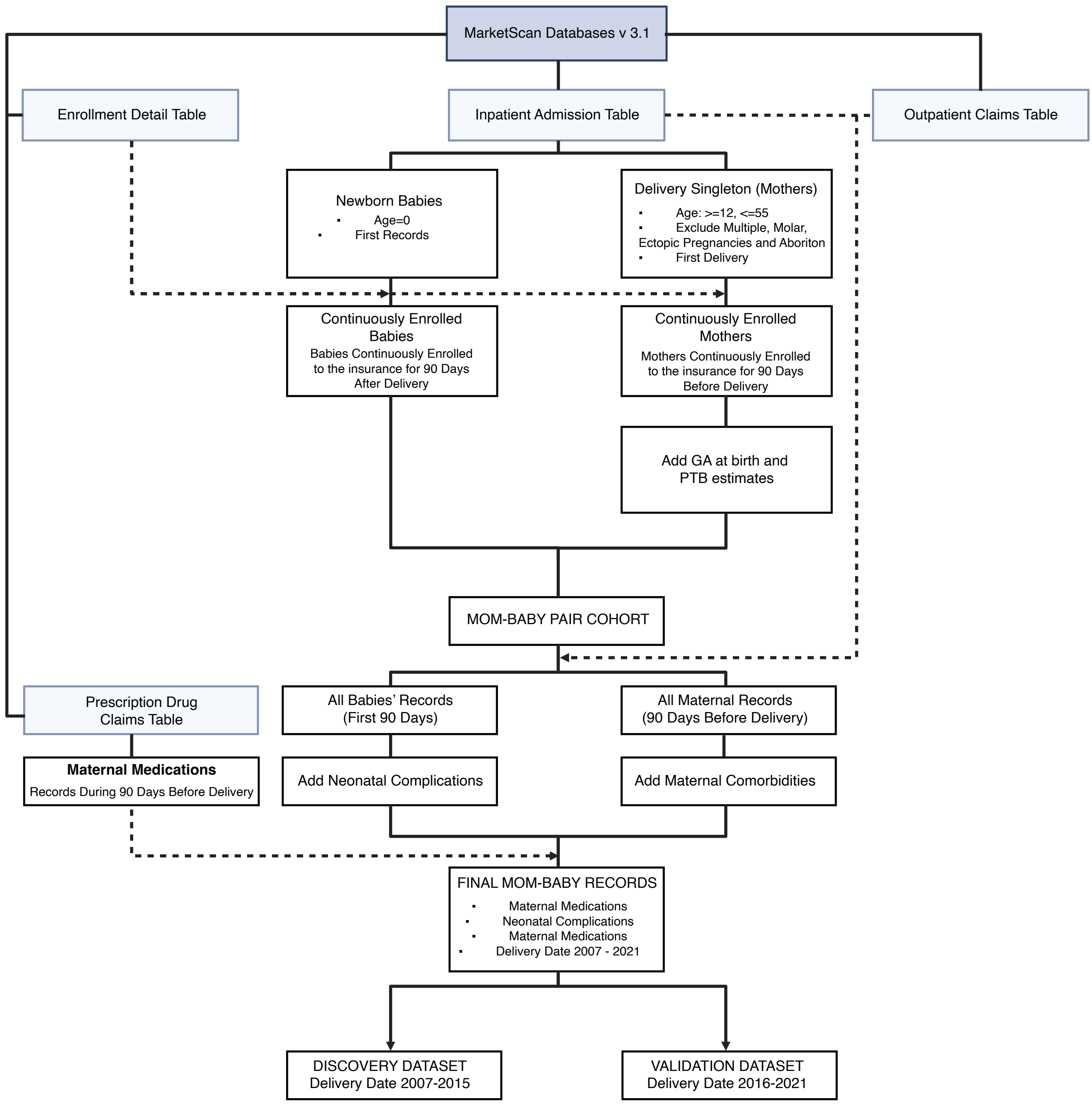

Supplementary Figure 1. Flow Chart of the Data Construction Overview. This figure illustrates the high-level process of data cleaning and preparation for analysis derived from the Merative™ Marketscan® Commercial Database.

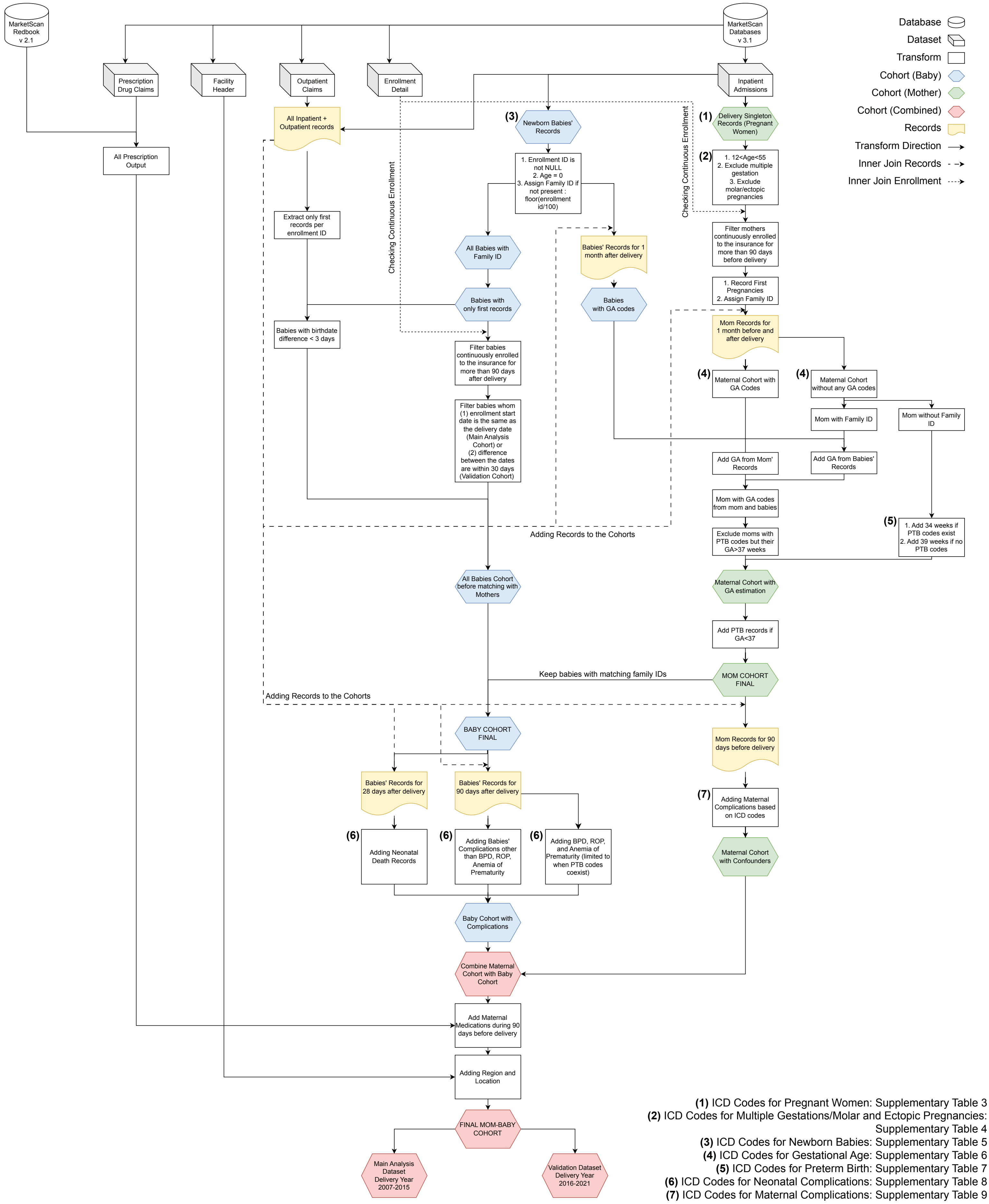

(1) ICD Codes for Pregnant Women: Supplementary Table 3
(2) ICD Codes for Multiple Gestations/Molar and Ectopic Pregnancies: Supplementary Table 4
(3) ICD Codes for Newborn Babies: Supplementary Table 5
(4) ICD Codes for Gestational Age: Supplementary Table 6
(5) ICD Codes for Preterm Birth: Supplementary Table 7
(6) ICD Codes for Neonatal Complications: Supplementary Table 8
(7) ICD Codes for Maternal Complications: Supplementary Table 9

Supplementary Figure 2. Comprehensive Flowchart Detailing Data Construction. This figure details the extensive process of data cleaning and preparation undertaken using the Merative™ Marketscan® Commercial Database. It expands upon the methodologies outlined in Supplementary Figure 1, providing clarity on the data preparation steps for the study. The flowchart depicts the procedural actions executed to assemble and refine the dataset, leading to the creation of a table that encapsulates maternal medication use, maternal comorbidities, 6 other attributes (maternal age, gestational age at delivery, preterm birth, baby's gender, and the region and geographic location of the healthcare facility), and neonatal complications. This offers a thorough perspective at the individual patient level and serves to enhance the foundational information displayed in Figure 1(a).

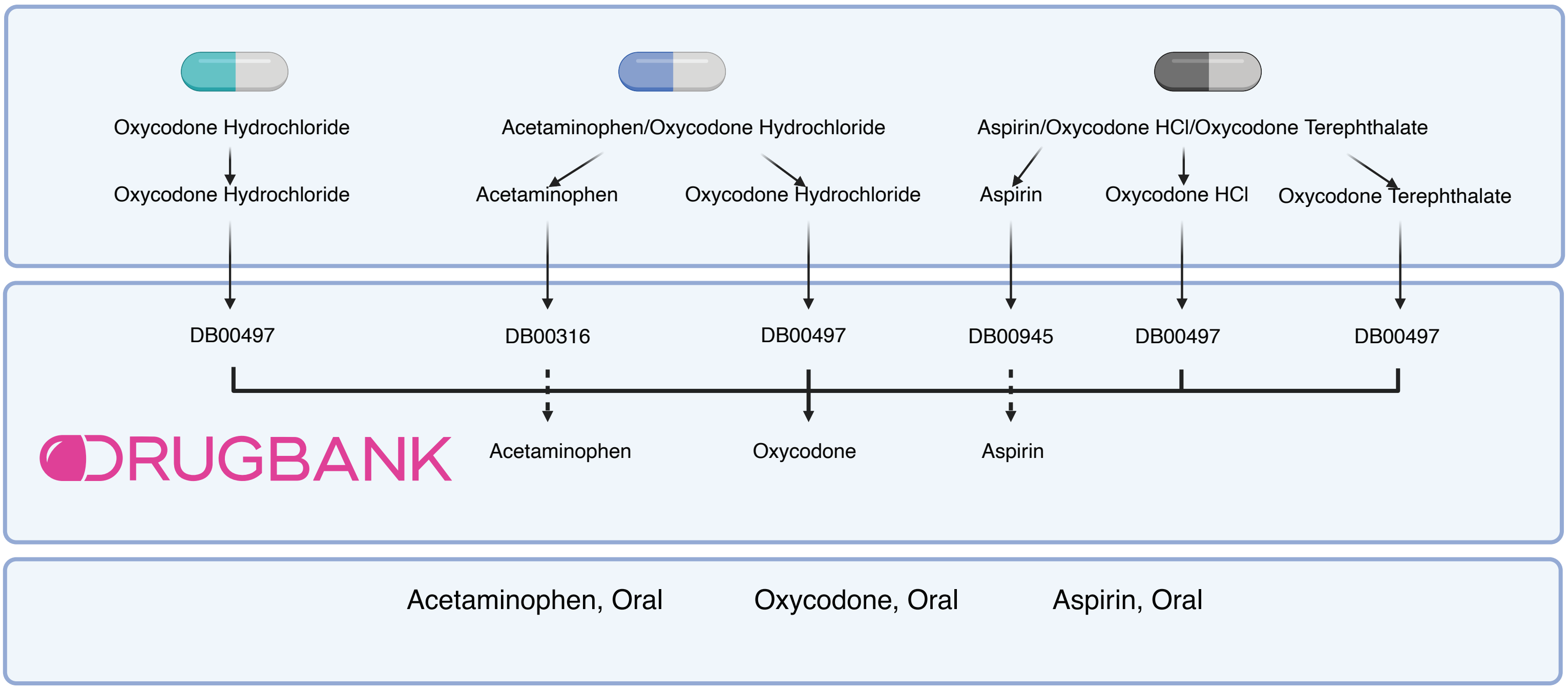

Supplementary Figure 3. Medication Name Harmonization. This figure outlines the process employed to clean up and harmonized maternal medication data. 1) Maternal medication with generic names was divided at the compound level. 2) Identical medications with varying names were consolidated and harmonized by employing DrugBank IDs along with their common names. 3) Lastly, information regarding the route of administration was integrated into these standardized names. Created in BioRender. Kim, Y. (2026) https://BioRender.com/mdtgmxz.

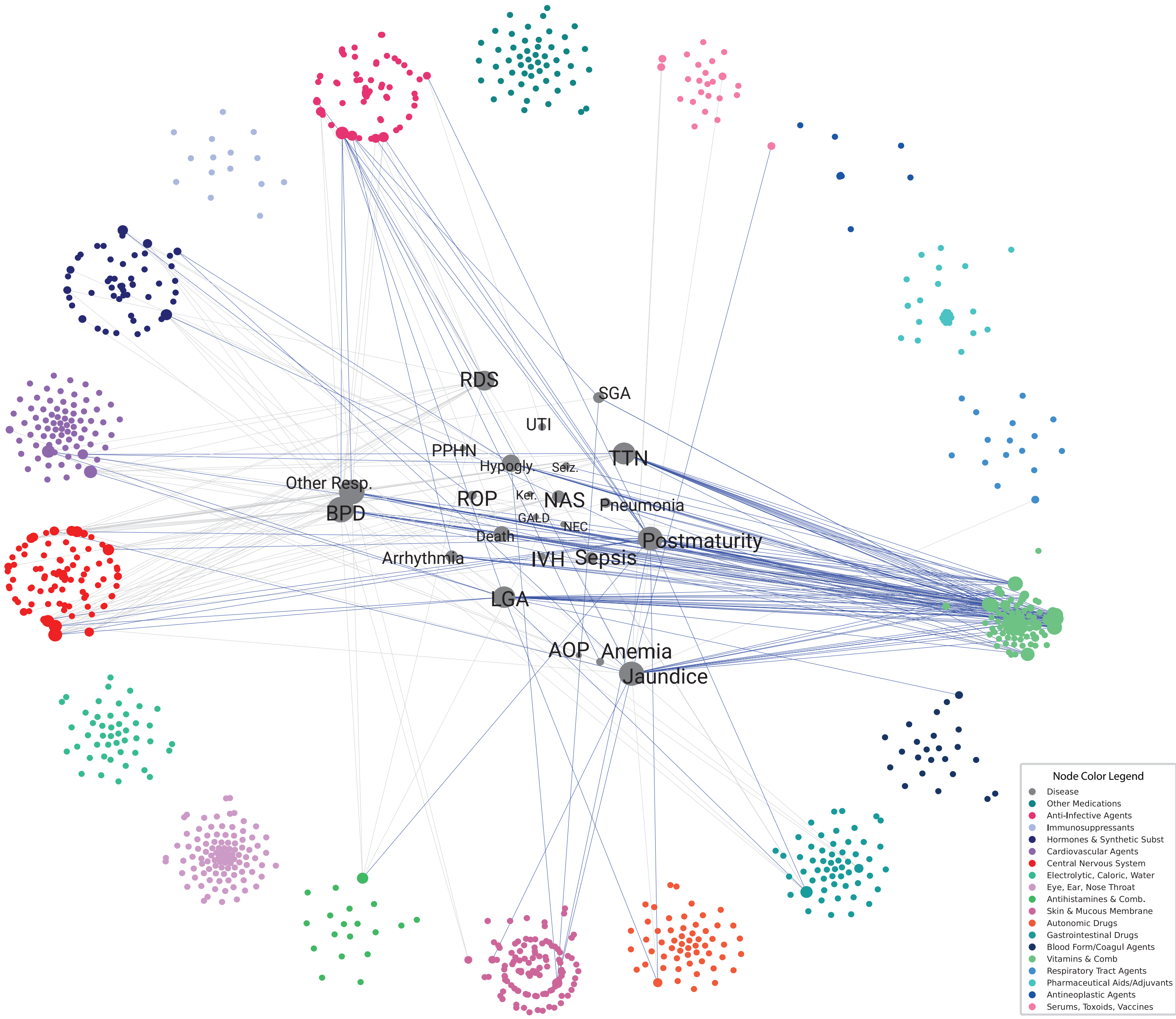

Supplementary Figure 4. Network Graph of the Adjusted Odds Ratios Between Maternal Medications and Neonatal Complications. Network graph depiction of adjusted odds ratios (aORs) between maternal medications and neonatal complications. aORs are calculated using LassoNoExp, a method that selects confounders by penalizing the excess of potential confounders for each medication-disease pair. Each node in the network graph represents either a maternal medication or a neonatal complication, consistent with the representation in Figure 2. Edges between nodes indicate statistically significant aORs, differentiated from unadjusted odds ratios presented Figure 2. PregMedNet identifies 261 statistically significant adjusted odds ratios (aORs) after adjustment, involving the associations of 68 maternal medications and 19 neonatal complications. Out of the total 261 edges, the 117 edges representing aORs > 1, indicating an increased risk association, are shown as gray edges, while the 144 edges representing aORs < 1, indicating a reduced risk association, are colored blue. The abbreviations for disease nodes are as defined in Figure 2.

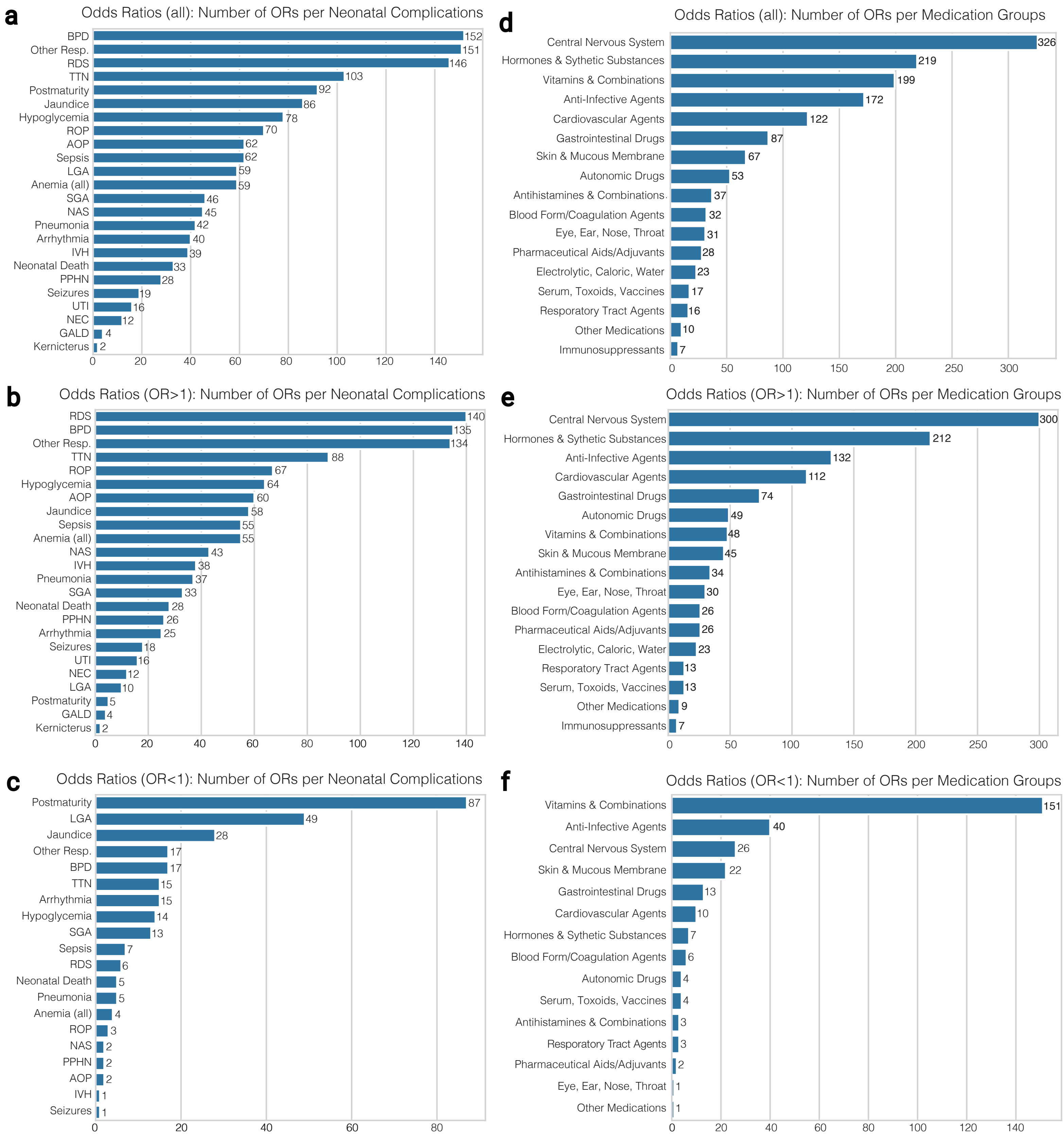

Supplementary Figure 5. Distribution of Unadjusted Odds Ratios (ORs) across Neonatal Complications and Maternal Medication Groups. Bar graphs showing the count of statistically significant ORs. (a) The number of all statistically significant odds ratios associated with neonatal complications. BPD showed the greatest number of significant associations, followed by other respiratory disorders, RDS, TTN, and Postmaturity. (b) The number of statistically significant increased risk associations (OR >1) associated with neonatal complications. RDS showed the most increased risk associations, followed by BPD, other respiratory disorders, TTN, and ROP. (c) The number of statistically significant reduced risk associations (OR <1) associated with neonatal complications. Postmaturity has the most reduced risk associations, followed by LGA and Jaundice. (d) The number of all significant ORs categorized by maternal medication groups. Medications in the Central Nervous System showed the most significant associations, followed by Hormones & Synthetic substances, Vitamins & Combinations, and Anti-Infective Agents. (e) The number of significant increased risk associations (OR >1) categorized by maternal medication groups. Medications in the Central Nervous System show the most increased risk associations, followed by Hormones & Synthetic Substances, Anti-Infective Agents, and Cardiovascular Agents. (f) The number of significant reduced risk associations (OR <1) categorized by maternal medication groups. The Vitamins & Combinations group shows the most reduced risk associations, followed by Anti-Infective Agents.

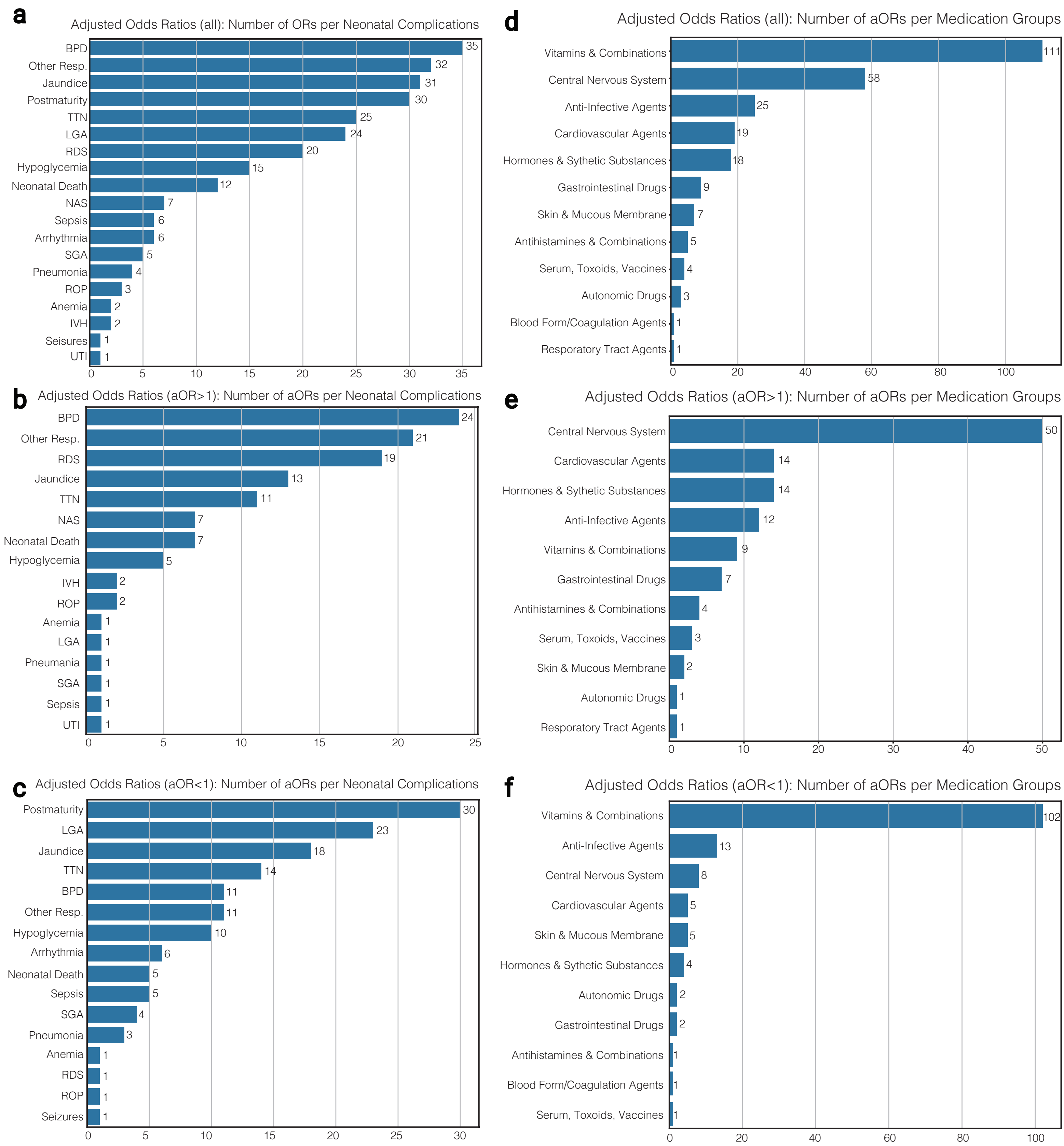

Supplementary Figure 6. Distribution of Adjusted Odds Ratios (aORs) across Neonatal Complications and Maternal Medication Groups. (a) The number of all statistically significant aORs associated with neonatal complications. BPD showed the most significant associations, followed by other respiratory disorders, Jaundice, Postmaturity, and TTN. (b) The number of statistically significant increased risk associations (aORs >1) associated with neonatal complications. BPD showed the most increased risk associations, followed by other respiratory disorders, RDS, Jaundice, and TTN. (c) The number of statistically significant reduced risk associations (aORs <1) associated with neonatal complications. Postmaturity has the most reduced risk associations followed by LGA and Jaundice. (d) The number of all significant aORs categorized by maternal medication groups. Medications in the Vitamins & Combinations showed the most significant associations, followed by Central Nervous System and Anti-Infective Agents. (e) The number of significant increased risk associations (aORs >1) categorized by maternal medication groups. Medications in the Central Nervous System show the most increased risk associations, followed by Cardiovascular Agents, Hormones & Synthetic Substances, and Anti-Infective Agents. (f) The number of significant reduced risk associations (aORs <1) categorized by maternal medication groups. The Vitamins & Combinations group shows the most reduced risk associations, followed by Anti-Infective Agents.

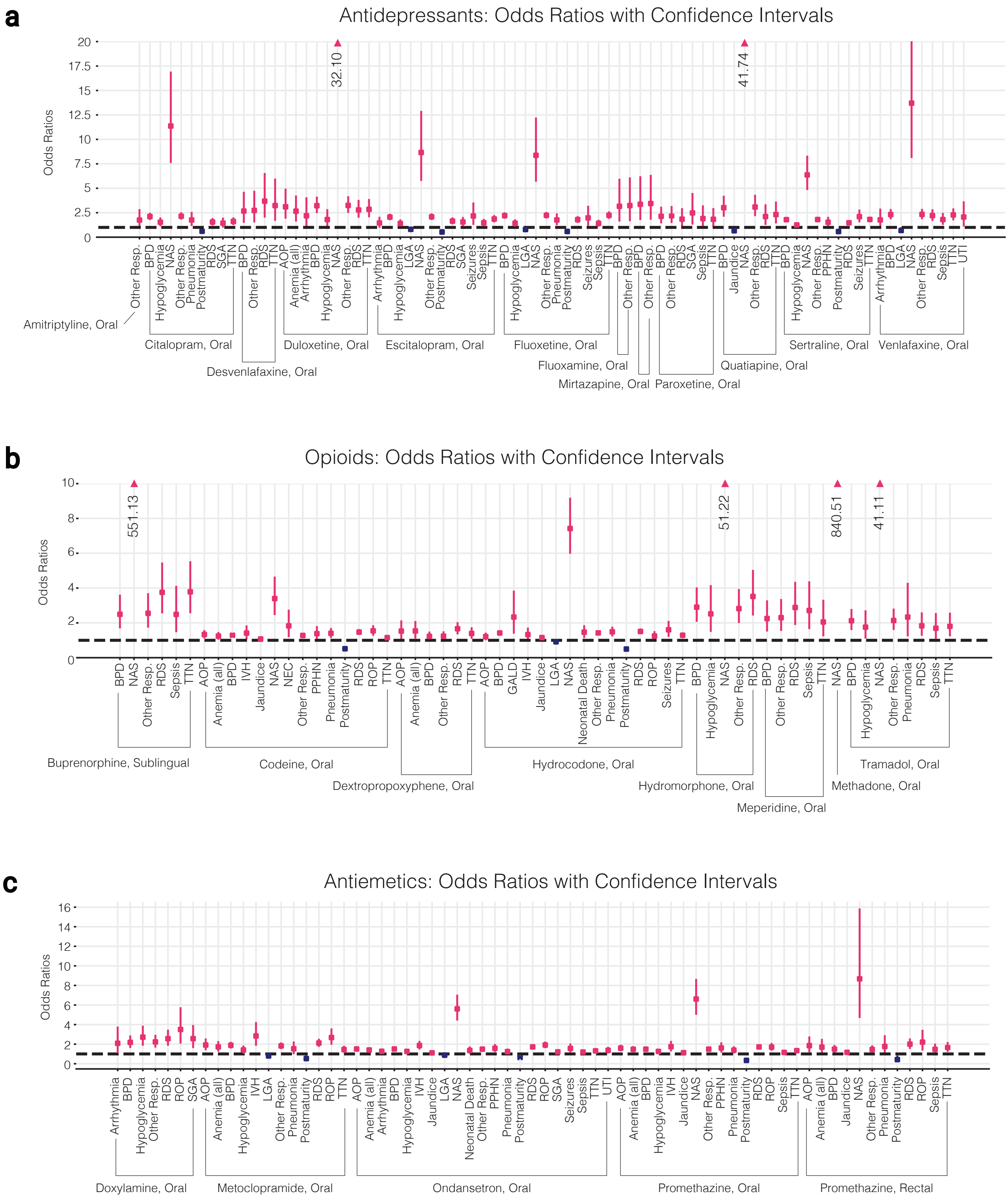

a
Antidepressants: Odds Ratios with Confidence Intervals
Odds Ratios
0
2.5
5
7.5
10
12.5
15
17.5
20
32.10
41.74
Other Resp. BPD Hypoglycemia NAS Other Resp. Pneumonia Postmaturity RDS SGA TTN BPD Other Resp. RDS TTN AOP Anemia (all) Arrhythmia BPD Hypoglycemia NAS Other Resp. RDS TTN Arrhythmia BPD Hypoglycemia LGA NAS Other Resp. Postmaturity RDS SGA Seizures Sepsis TTN BPD Hypoglycemia LGA NAS Other Resp. Pneumonia Postmaturity RDS Seizures Sepsis TTN BPD Other Resp. BPD Other Resp. BPD Other Resp. RDS SGA Sepsis TTN BPD Jaundice NAS Other Resp. RDS TTN BPD Hypoglycemia NAS Other Resp. PPHN Postmaturity RDS Seizures TTN Arrhythmia BPD LGA NAS Other Resp. RDS Sepsis TTN UTI
Amitriptyline, Oral
Citalopram, Oral
Desvenlafaxine, Oral
Duloxetine, Oral
Escitalopram, Oral
Fluoxetine, Oral
Fluoxamine, Oral
Mirtazapine, Oral
Paroxetine, Oral
Quatiapine, Oral
Sertraline, Oral
Venlafaxine, Oral
b
Opioids: Odds Ratios with Confidence Intervals
Odds Ratios
0
2
4
6
8
10
551.13
51.22
840.51
41.11
BPD NAS Other Resp. RDS Sepsis TTN AOP Anemia (all) BPD IVH Jaundice NAS NEC Other Resp. PPHN Pneumonia Postmaturity RDS ROP TTN AOP Anemia (all) BPD Other Resp. RDS TTN AOP BPD GALD IVH Jaundice LGA NAS Neonatal Death Other Resp. Pneumonia Postmaturity RDS ROP Seizures TTN BPD Hypoglycemia NAS Other Resp. RDS BPD Other Resp. RDS Sepsis TTN NAS BPD Hypoglycemia NAS Other Resp. Pneumonia RDS Sepsis TTN
Buprenorphine, Sublingual
Codeine, Oral
Dextropropoxyphene, Oral
Hydrocodone, Oral
Hydromorphone, Oral
Meperidine, Oral
Methadone, Oral
Tramadol, Oral
c
Antiemetics: Odds Ratios with Confidence Intervals
Odds Ratios
0
2
4
6
8
10
12
14
16
Arrhythmia BPD Hypoglycemia Other Resp. RDS ROP SGA AOP Anemia (all) BPD Hypoglycemia IVH LGA Other Resp. Pneumonia Postmaturity RDS ROP TTN AOP Anemia (all) Arrhythmia BPD Hypoglycemia IVH Jaundice LGA NAS Neonatal Death Other Resp. PPHN Pneumonia Postmaturity RDS ROP SGA Seizures Sepsis TTN UTI AOP Anemia (all) BPD Hypoglycemia IVH Jaundice NAS Other Resp. PPHN Pneumonia Postmaturity RDS ROP Sepsis TTN AOP Anemia (all) BPD Jaundice NAS Other Resp. Pneumonia Postmaturity RDS ROP Sepsis TTN
Doxylamine, Oral
Metoclopramide, Oral
Ondansetron, Oral
Promethazine, Oral
Promethazine, Rectal

d

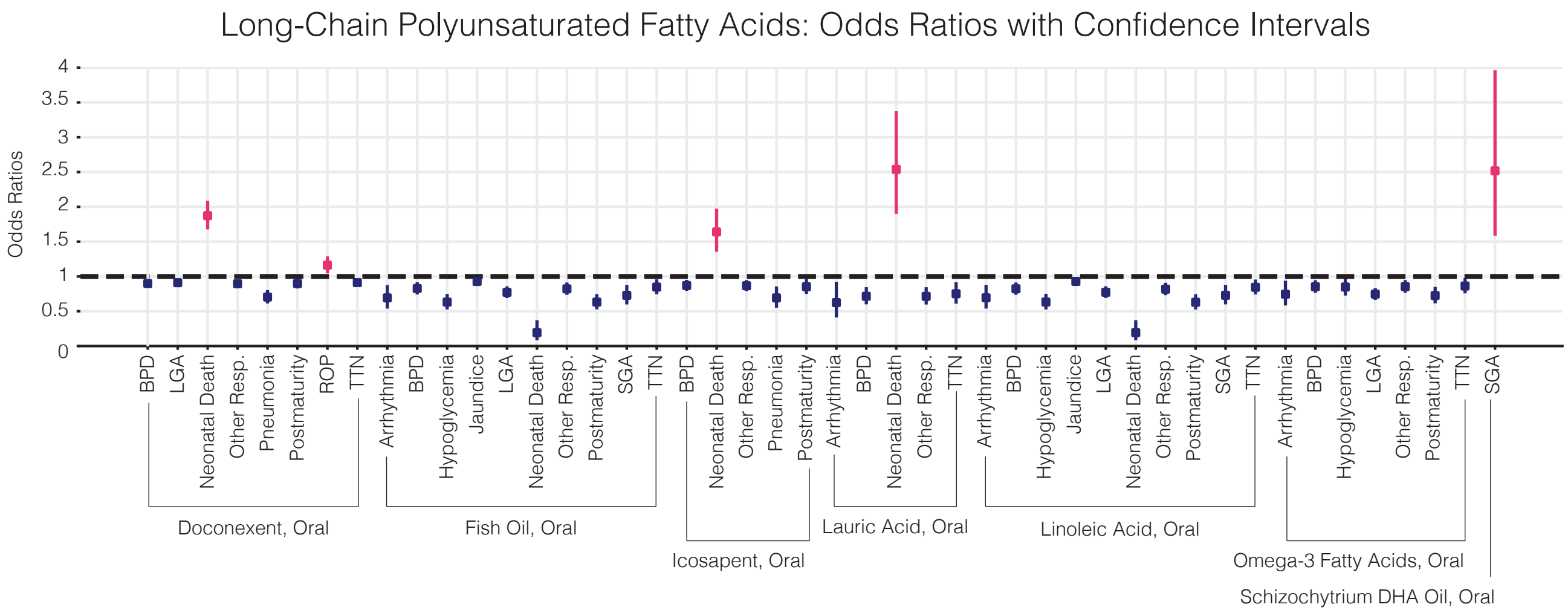

e

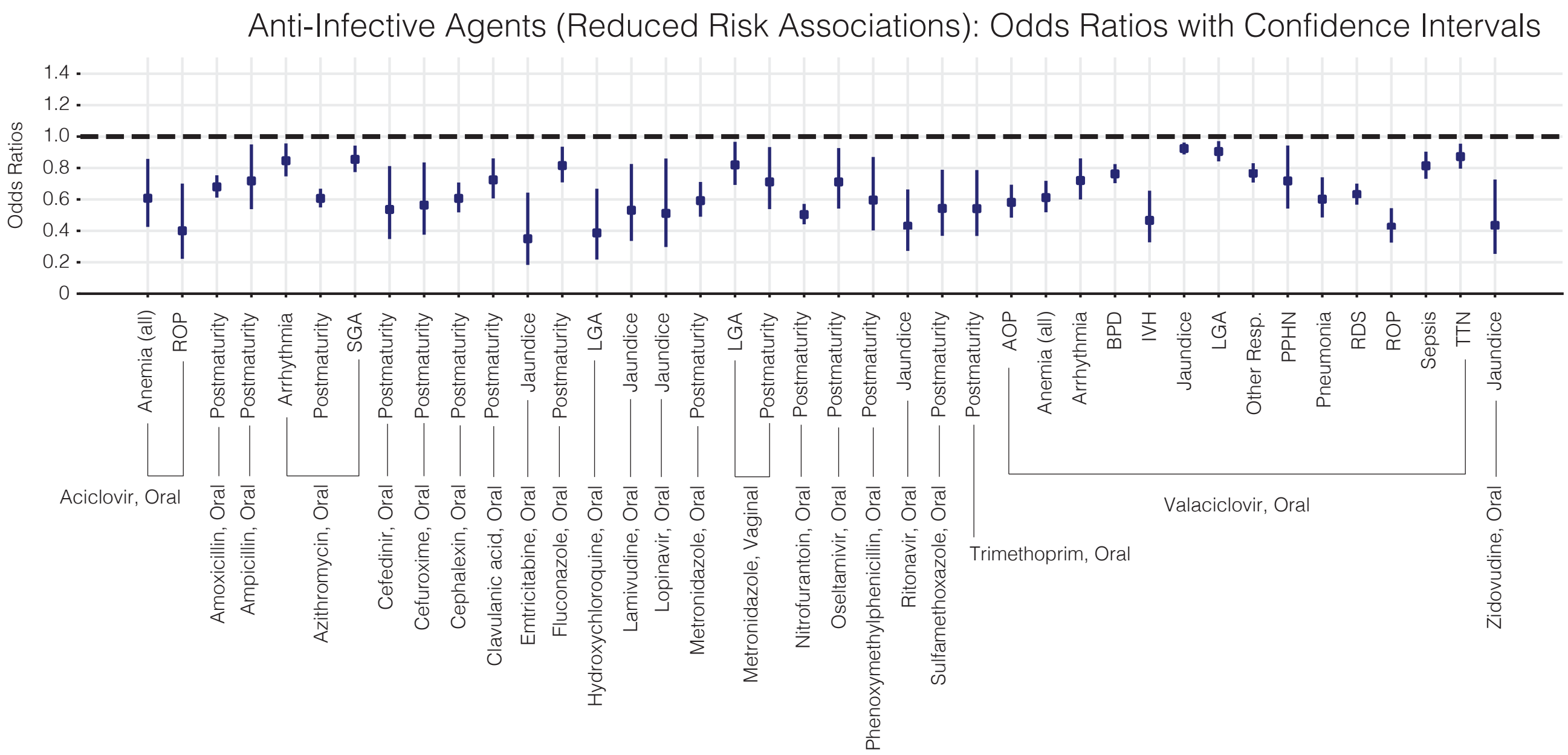

Supplementary Figure 7. Unadjusted odds ratios for five different medication groups. The figure shows statistically significant unadjusted odds ratios (ORs) and their 95% confidence intervals (CIs) across five medication groups. Each dot represents an OR, and error bars represent 95% CIs. Associations with ORs exceeding the y-axis range are indicated by triangles and annotated with exact OR values; corresponding CIs extending beyond the axis range are truncated at the plot boundary. Results are derived from the discovery cohort (n = 817,402 mother–newborn dyads) from the Merative™ Marketscan® Commercial Database. Magenta indicates OR > 1 (increased risk), whereas navy blue indicates OR < 1 (reduced risk). Exact numerical values, including 95% CIs and p-values, are available at pregmednet.stanford.edu. (a) Antidepressants. (b) Opioids. (c) Antiemetics. (d) Long-chain polyunsaturated fatty acids (vitamin and combination group). (e) Reduced-risk associations (OR < 1) in anti-infective groups.

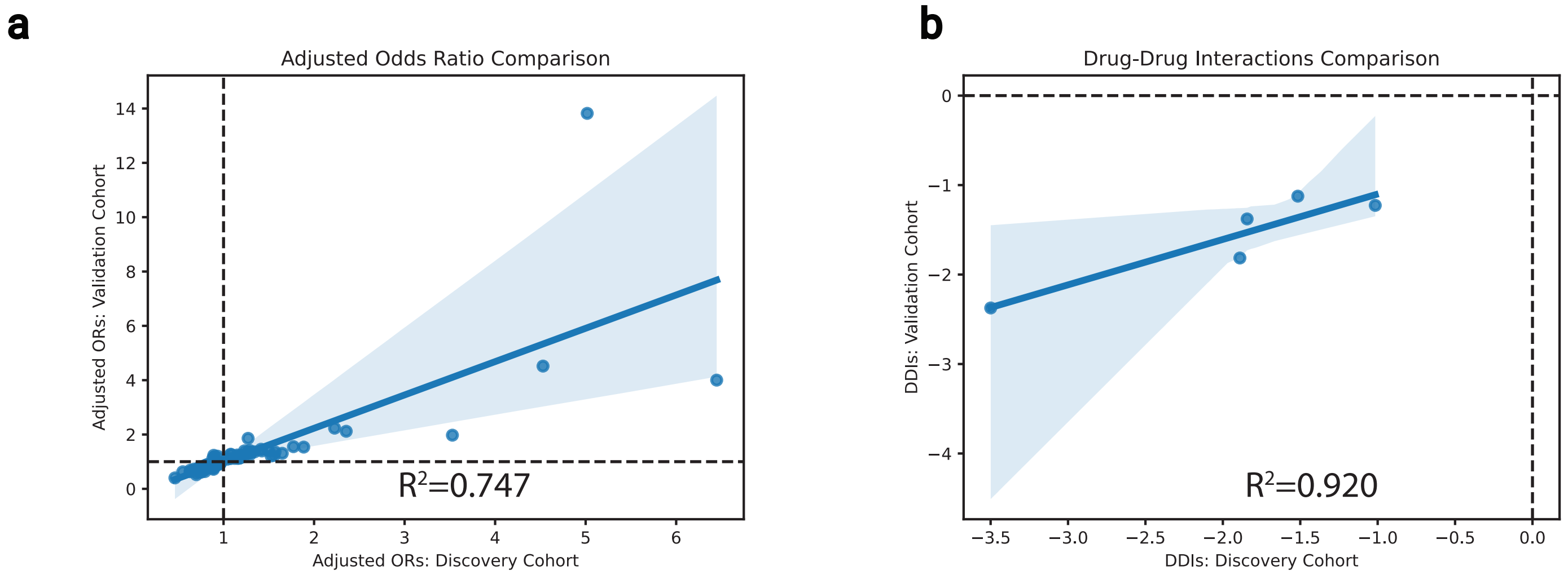

Supplementary Figure 8. Correlation between results from the discovery and validation cohorts. Scatterplots show matched effect estimates (adjusted odds ratios or drug-drug interactions) between the discovery and validation cohorts for the same maternal medication–neonatal complication pairs. Pearson correlation was assessed using a two-sided test. (a) Scatterplot of significant adjusted odds ratios (aORs) in the discovery cohort (x-axis) and the validation cohort (y-axis). In total, 81 aORs are matched between the two cohorts, out of 261 from the discovery cohort and 167 from the validation cohort. Pearson correlation analysis yields $R^2 = 0.747$ and $P = 1.246 \times 10^{-15}$. (b) Scatterplot of significant drug–drug interactions (β_3) in the discovery cohort (x-axis) and the validation cohort (y-axis). In total, 5 adjusted DDIs are matched between the two cohorts, out of 72 from the discovery cohort and 15 from the validation cohort. Pearson correlation analysis yields $R^2 = 0.920$ and $P = 2.682 \times 10^{-2}$.

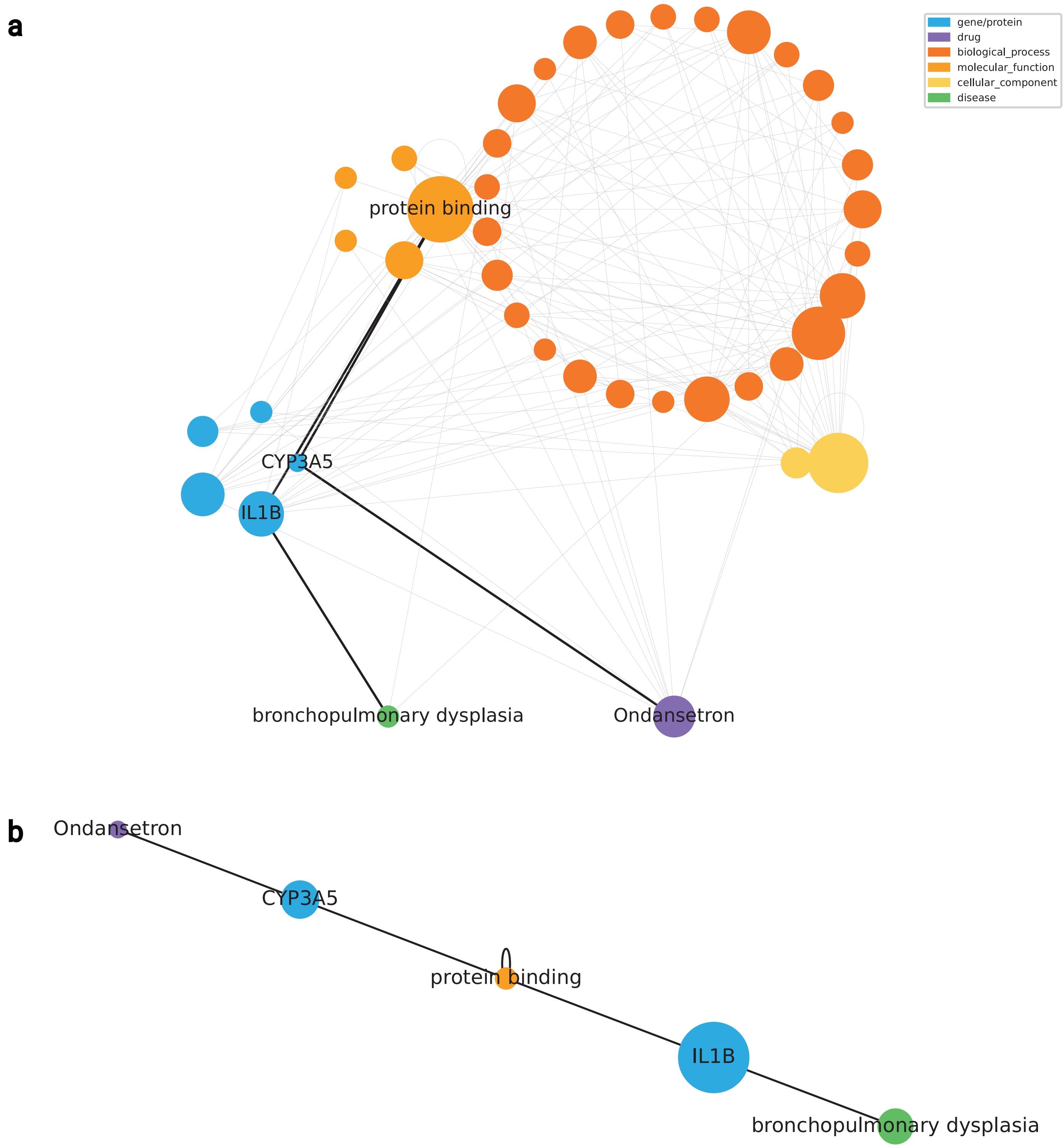

__Supplementary Figure 9. Potential Underlying Mechanisms of Ondansetron Effects on Bronchopulmonary Dysplasia with Inclusion of All Biological Nodes.__ (a) Hypothesized associations between maternal medications and neonatal complications are generated by combining PregMedNet results with a Knowledge Graph. This is an expanded version of Figure 6. While Figure 6 includes only protein/gene nodes, this subgraph depiction includes all biological node types as intermediate nodes between the disease and the medication of interest. The figure depicts the shortest paths between bronchopulmonary dysplasia and ondansetron. The subgraph contains 60 four-hop paths and 41 nodes, including the medication and disease nodes. (b) The path involving IL-1β and CYP (CYP3A5) is also found in this subgraph, but IL-1β and CYP are connected by a ‘protein binding’ node.

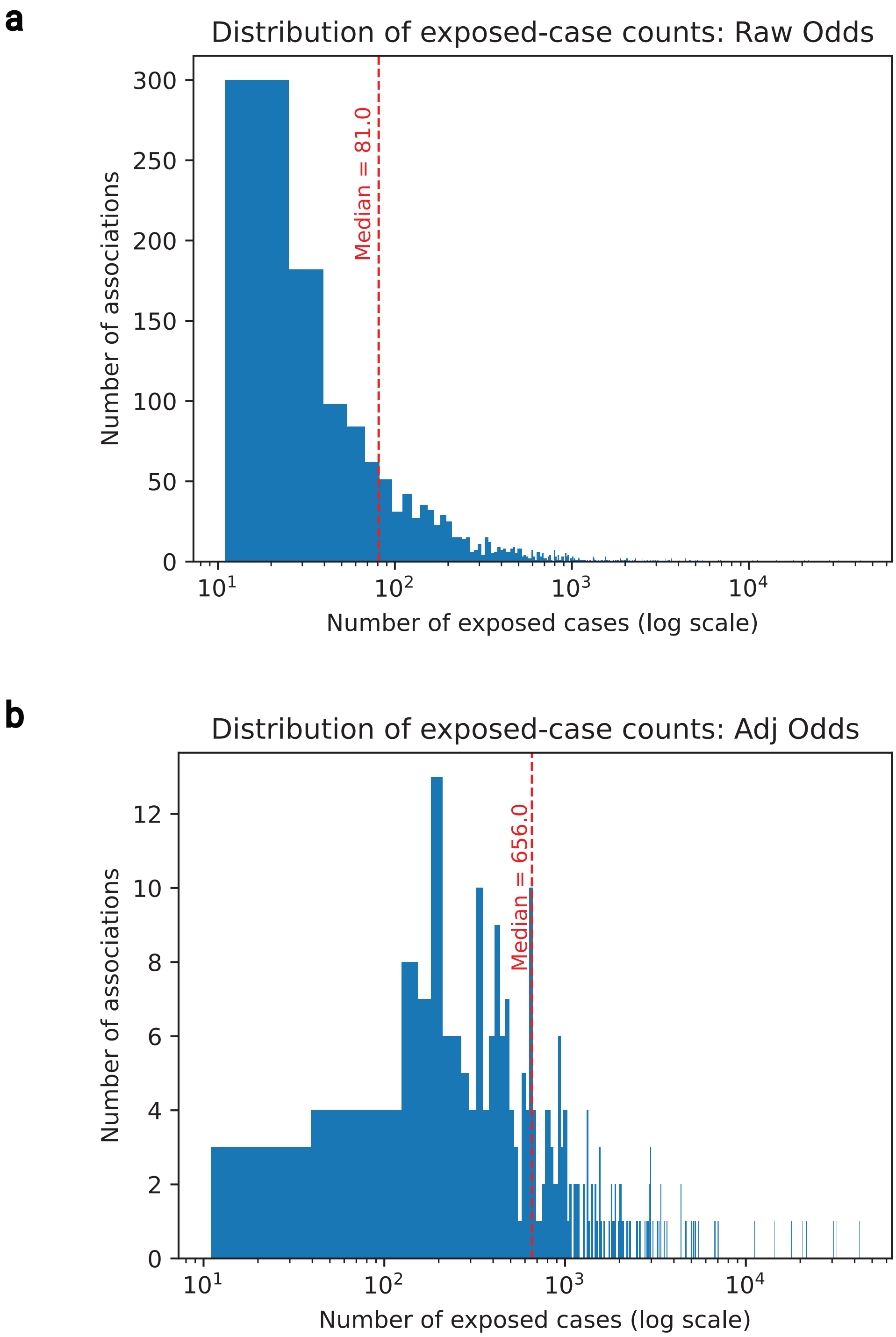

Supplementary Figure 10. Distributions of exposed-case counts for signification associations. Histograms showing the distribution of the number of exposed cases among significant associations for (a) raw odds ratios (ORs) and (b) adjusted odds ratios (aORs). The x-axis represents the number of exposed cases on a logarithmic scale, and the y-axis represents the number of associations. Red dashed lines indicate the median number of exposed cases for each analysis.
