# Supplementary material for: PregMedNet: Multifaceted maternal medication impacts on neonatal complications
Source: Nat Commun. 2026 Jul 10;17:8526. doi: 10.1038/s41467-026-75000-0 (PMC13483950; doi:10.1038/s41467-026-75000-0)
Supplement: Supplementary file 2 [file 41467_2026_75000_MOESM2_ESM.pdf]

Description of Additional Supplementary Files

Supplementary Data 1
Description: Maternal Age, Gestational Age, Preterm Birth

Supplementary Data 2
Description: Prevalence of Diseases (Mother and Baby)

Supplementary Data 3
Description: Unavailable demographic and behavioral variables and their impact on association estimates

Supplementary Data 4
Description: Distribution of Maternal Medications in the discovery cohort according to Australian categories for prescribing medicine in pregnancy (AU TGA)

Supplementary Data 5
Description: ICD/CPT Codes for Pregnancy (Delivery Singleton)

Supplementary Data 6
Description: ICD Codes for Multiple Gestations and Ectopic/Molar Pregnancies

Supplementary Data 7
Description: ICD Codes for Newborn Babies

Supplementary Data 8
Description: ICD Code List for 68 Maternal Comorbidities

Supplementary Data 9
Description: ICD Code List for 24 Neonatal Complications

Supplementary Data 10
Description: ICD Codes for Gestational Age

Supplementary Data 11
Description: ICD Codes for Preterm Birth
