# Supplementary material for: PregMedNet: Multifaceted maternal medication impacts on neonatal complications
Source: Nat Commun. 2026 Jul 10;17:8526. doi: 10.1038/s41467-026-75000-0 (PMC13483950; doi:10.1038/s41467-026-75000-0)
Supplement: Supplementary file 3 [file 41467_2026_75000_MOESM4_ESM.pdf]

nature portfolio

Corresponding author(s): Yeasul Kim
Nima Aghaeepour

Last updated by author(s): May 7, 2026

Reporting Summary

Nature Portfolio wishes to improve the reproducibility of the work that we publish. This form provides structure for consistency and transparency in reporting. For further information on Nature Portfolio policies, see our Editorial Policies and the Editorial Policy Checklist.

Statistics

For all statistical analyses, confirm that the following items are present in the figure legend, table legend, main text, or Methods section.

n/a	Confirmed	
☐	☒	The exact sample size (n) for each experimental group/condition, given as a discrete number and unit of measurement
☐	☒	A statement on whether measurements were taken from distinct samples or whether the same sample was measured repeatedly
☐	☒	The statistical test(s) used AND whether they are one- or two-sided<br>*Only common tests should be described solely by name; describe more complex techniques in the Methods section.*
☐	☒	A description of all covariates tested
☐	☒	A description of any assumptions or corrections, such as tests of normality and adjustment for multiple comparisons
☐	☒	A full description of the statistical parameters including central tendency (e.g. means) or other basic estimates (e.g. regression coefficient) AND variation (e.g. standard deviation) or associated estimates of uncertainty (e.g. confidence intervals)
☐	☒	For null hypothesis testing, the test statistic (e.g. F, t, r) with confidence intervals, effect sizes, degrees of freedom and P value noted<br>*Give P values as exact values whenever suitable.*
☒	☐	For Bayesian analysis, information on the choice of priors and Markov chain Monte Carlo settings
☐	☒	For hierarchical and complex designs, identification of the appropriate level for tests and full reporting of outcomes
☐	☒	Estimates of effect sizes (e.g. Cohen's d, Pearson's r), indicating how they were calculated

Our web collection on statistics for biologists contains articles on many of the points above.

Software and code

Policy information about availability of computer code

Data collection	Data collected through Merative™ Marketscan® Commercial Database and DrugBank database.
Data analysis	Data analysis was performed using Python 3.10, with key libraries including scikit-learn (v1.2.2), statsmodels (v0.13.5), optuna (v3.4.0), bokeh (v2.4.3), and NetworkX (v3.2.1). Custom algorithms and scripts central to this study are available at https://github.com/yerakim824/PregMedNet/, and all findings are accessible through an interactive website at https://pregmednet.stanford.edu/.

For manuscripts utilizing custom algorithms or software that are central to the research but not yet described in published literature, software must be made available to editors and reviewers. We strongly encourage code deposition in a community repository (e.g. GitHub). See the Nature Portfolio guidelines for submitting code & software for further information.

Data

Policy information about availability of data

All manuscripts must include a data availability statement. This statement should provide the following information, where applicable:

- Accession codes, unique identifiers, or web links for publicly available datasets
- A description of any restrictions on data availability
- For clinical datasets or third party data, please ensure that the statement adheres to our policy

The data that support the findings of this study are not publicly available due to restrictions imposed by the data provider and the data use agreement. The dataset used in this study is the Merative™ Marketscan® Commercial Database, a proprietary dataset containing de-identified health information derived from private insurance claims.

The data use agreement prohibits the redistribution of both raw and processed data. Access to the data is available to qualified entities (including academic, nonprofit, governmental, and commercial institutions) through purchase and a formal data use agreement with Merative™.
Requests for access should be directed to the Merative™ Product Support Team (productsupport@merative.com). Additional information regarding data access and licensing is available at: https://www.merative.com/documents/brief/marketscan-explainer-general. The time required to obtain access may vary depending on the contracting process. The data are available for the duration of the licensing period.
To facilitate reproducibility, detailed descriptions of data processing and analysis are provided in the Methods section, and the code used in this study is publicly available at: https://github.com/yerakim824/PregMedNet.

Research involving human participants, their data, or biological material

Policy information about studies with human participants or human data. See also policy information about sex, gender (identity/presentation), and sexual orientation and race, ethnicity and racism.

Reporting on sex and gender	There are a total of 1,190,257 mother-baby dyads included. All mothers are female. Among the babies, 614,695 (51.6%) are male, and 575,562 (48.4%) are female. Sex was determined based on assignment at birth as recorded in the claims data.
Reporting on race, ethnicity, or other socially relevant groupings	Race, ethnicity, and other socially relevant information is unavailable in Merative™ Marketscan® Commercial Database.
Population characteristics	The pregnancy cohort and their corresponding babies' cohort (mother-baby dyads) were created by retrospectively extracting the cohort from the Merative™ MarketScan® Commercial Database using the International Classification of Diseases, Ninth Revision (ICD-9), ICD-10, and diagnosis-related group (DRG) codes from 2007 to 2021. Women aged 12–55 years with singleton, first pregnancies were included, while molar and ectopic pregnancies, as well as spontaneous and induced abortions, were excluded. Their corresponding newborn cohort was identified using newborn-related ICD-9 and ICD-10 codes from 2007 to 2021. To ensure comprehensive data capture, only mothers and babies continuously enrolled in the insurance plan during the observation period were included.
Recruitment	The maternal and neonatal information is extracted from retrospective, de-identifed claims database, Merative™ Marketscan® Commercial Database.
Ethics oversight	This research complies with all relevant ethical regulations. This study was reviewed and approved by the institutional review board (IRB) at Stanford University (no. 39225). The requirement for informed consent was waived because the study used de-identified, retrospective data and posed minimal risk to participants, in accordance with applicable regulations. No participant compensation was provided.

Note that full information on the approval of the study protocol must also be provided in the manuscript.

Field-specific reporting

Please select the one below that is the best fit for your research. If you are not sure, read the appropriate sections before making your selection.

☒ Life sciences ☐ Behavioural & social sciences ☐ Ecological, evolutionary & environmental sciences

For a reference copy of the document with all sections, see nature.com/documents/nr-reporting-summary-flat.pdf

Life sciences study design

All studies must disclose on these points even when the disclosure is negative.

Sample size	Our study consists of two parts. The first part involves mother-baby dyads extracted from a retrospective, de-identified claims dataset, which is used to generate initial hypotheses. This dataset includes 1,190,257 mother-baby dyads, totaling 2,380,514 individuals. The second part of the study involves a mouse model to support a novel hypothesis generated from the first part. This model includes 13 maternal mice and 44 pups.
Data exclusions	No data were excluded.
Replication	The mother-baby dyads were divided into two groups based on birth date. Babies delivered between 2007 and 2015 were included in the discovery cohort to generate the initial hypothesis, while those delivered between 2016 and 2021 formed the validation cohort to assess hypothesis replication. The Pearson correlation coefficients between the results from the two cohorts were 0.972 (odds ratios), 0.747 (adjusted odds ratios), and 0.920 (drug-drug interactions), ensuring the reproducibility of our findings.<br><br>Additionally, the mouse model experiment replicated one of the hypotheses, demonstrating increased odds of bronchopulmonary dysplasia in babies born to mothers who took ondansetron. To test medication safety, 24 mice were used for experimental testing, while the remaining 20 mice served as controls.
Randomization	There is no group allocation in this study.
Blinding	There is no group allocation in this study.

Reporting for specific materials, systems and methods

We require information from authors about some types of materials, experimental systems and methods used in many studies. Here, indicate whether each material, system or method listed is relevant to your study. If you are not sure if a list item applies to your research, read the appropriate section before selecting a response.

Materials & experimental systems

n/a	Involved in the study	
☒	☐	Antibodies
☒	☐	Eukaryotic cell lines
☒	☐	Palaeontology and archaeology
☐	☒	Animals and other organisms
☒	☐	Clinical data
☒	☐	Dual use research of concern
☒	☐	Plants

Methods

n/a	Involved in the study	
☒	☐	ChIP-seq
☒	☐	Flow cytometry
☒	☐	MRI-based neuroimaging

Animals and other research organisms

Policy information about studies involving animals; ARRIVE guidelines recommended for reporting animal research, and Sex and Gender in Research

Laboratory animals	Female and male C57BL/6J mice (Mus musculus; genetic background: C57BL/6) were purchased from The Jackson Laboratory and maintained in-house under standard housing conditions with a 12-hour light/12-hour dark cycle, ambient temperature of 18–23°C, and humidity of 40–60%. A total of 20 female and 10 male C57BL/6J mice aged 8–12 weeks were used for timed matings. For experiments, 9 pregnant dams were used, with 4 dams receiving PBS in the control group (resulting in n=20 control pups) and 5 dams receiving ondansetron in the experimental group (resulting in n=24 ondansetron pups). Following the lung selection and subsequent quality control of lung specimens, 9 controls and 12 ondansetron-exposed pups were included in the final quantitative analysis of the Mean Linear Intercept (MLI). Sex was not considered in the study design because neonatal mice were used in the study, before sex could be accurately determined.
Wild animals	The study did not involve wild animals.
Reporting on sex	Sex was not considered in the study design because neonatal mice were used in the study, before sex could be accurately determined.
Field-collected samples	The study did not involve field-collected samples.
Ethics oversight	All animal experiments were approved by Stanford University's Institutional Animal Care and Use Committee (IACUC) under protocol no. 33897, and were conducted in accordance with relevant institutional and national guidelines and regulations.

Note that full information on the approval of the study protocol must also be provided in the manuscript.

Plants

Seed stocks	No seed stocks or other plant materials was used in this study.
Novel plant genotypes	No seed stocks or other plant materials was used in this study.
Authentication	No seed stocks or other plant materials was used in this study.
