# Supplementary material for: PregMedNet: Multifaceted maternal medication impacts on neonatal complications
Source: Nat Commun. 2026 Jul 10;17:8526. doi: 10.1038/s41467-026-75000-0 (PMC13483950; doi:10.1038/s41467-026-75000-0)
Supplement: Supplementary file 4 [file 41467_2026_75000_MOESM5_ESM.pdf]

nature portfolio

Peer Review File

PregMedNet: Multifaceted Maternal Medication Impacts on Neonatal Complications

Corresponding Author: Professor Nima Aghaeepour

This file contains all reviewer reports in order by version, followed by all author rebuttals in order by version.

Version 0:

Reviewer comments:

Reviewer #1

(Remarks to the Author)
The manuscript provides a nice tool for predicting the maternal-neonatal medication toxicity and can contribute to advancing maternal medication safety.
Based on the results of their proposed model, the researchers have found a link between ondansetron and increased neonatal respiratory risks. To validate this outcome, the researchers have conducted an in vivo mice model. However, there are a few concerns regarding the in vivo experimental design and data analysis. The experimental design is not fully explained (e.g. statistical calculation for sample size) and a justification of dosing/timing is missing:

1- According to PregMedNet results, researchers have found significant links between orally administered ondansetron and neonatal respiratory complications. However, in their in vivo experiment, they chose to administer this drug intraperitoneally. What was the justification for choosing IP rout instead of oral gavage?

2- The average recommended dose of ondansetron in humans is 8 mg (not to exceed 16 mg). However, the researchers have used 5mg/kg b.w. in mice, which is relatively a higher dose compared to what is used in humans (ranging between 0.2 – 0.05 mg/kg b.w.). How do you justify the dose you chose?

3- Ondansetron is used to prevent nausea and vomiting caused by chemotherapies or certain surgeries. Aside from that, it might be used for severe pregnancy nausea during the first trimester. However, researchers observed a link between ondansetron administration during the last trimester and neonatal complications. Therefore, the prescription of this medication to this group of pregnant patients could be accompanied by other underlying diseases or in combination to other medications. Considering these information:
a. Is the proposed PregMedNet tool capable of incorporate the additional parameters, such as underlying medical conditions and multidrug administration, in this prediction?
b. Ondansetron is usually prescribed as a single prophylactic dose, or for maximum 1-2 days post chemotherapy or surgery. However, in the in vivo experiment researchers used daily IP injections for 5-6 days. How do you justify your exposure duration? (There are of course a few off-label dosages for pruritus treatment that longer administration is recommended. However, the duration of medication is not specified in PregMedNet results)

4- Was there any effect of ondansetron on the course of pregnancy, e.g. number of pups per litter or resorptions, other (clinical) symptoms in pregnant mice etc?

5- How was the sample size for this experiment calculated? Did you base the calculations on the number of pregnant mice or the pups? Since you collected samples from pups before weaning, did you consider each litter as an experimental unit, instead of individual pups? You had 20-24 pups from 4-5 litters, but you chose 9-12 mice for quantitative analysis. What was your criteria of choosing these mice? Was it a randomized selection, or did you select an average number of pups per litter? Regarding the statistical analysis performed in figure 5e, was it performed using all data points (135-180 images) or only on the average values per mouse?

(Remarks on code availability)
Evaluating the code is not within my expertise.

Reviewer #2

(Remarks to the Author)
Strengths
1.Clear Public Health Relevance and Timeliness

PregMedNet addresses a critical and growing public health need: the lack of robust evidence on the safety of medications used during pregnancy, particularly with respect to long-term outcomes. By integrating a wide range of high-quality data sources—including hospital records and claims data—the platform directly responds to urgent challenges in maternal and neonatal health. In addition, PregMedNet enables finer-grained investigation of maternal medication effects by retrieving drug information in a data-driven, compound-level manner and by distinguishing between routes of administration. This distinction allows the system to differentiate clinically distinct use cases for the same medication, such as topical betamethasone for skin inflammation versus intramuscular betamethasone for preventing preterm labor complications. Finally, the study examines the additive effects of concurrent maternal medication exposures on neonatal outcomes through a retrospective drug–drug interaction (DDI) analysis. To our knowledge, this represents the first large-scale, real-world, multi-institutional effort to systematically screen the effects of maternal drug combinations on neonatal complications.
2.Methodological Innovation
This project introduces several important methodological advances: (a) a systematic approach for identifying associations between maternal medication use and neonatal complications, (b) robust adjustment for confounding variables across numerous medication–disease pairs, (c) in vivo experimental validation supporting one of the data-driven hypotheses, and (d) the proposal of plausible mechanisms of action (MoA). A central contribution of this work is the implementation of a machine-learning–based framework for confounding adjustment
Points for considerations
1.Clarification of Twins and Other Multiple Births
The manuscript would benefit from a clearer justification for the exclusion of twins and other multiple births. Multiple gestations inherently carry higher and clinically distinct risks, that are not directly comparable to singleton pregnancies. The PregMedNet by it inherent approach could help close the gap of medications effects on multiple births.

2.First Pregnancy
Similarly, the decision to include only the first pregnancy per woman deserves further explanation.
3.Spontaneous abortion
The exclusion of spontaneous abortions also warrants additional justification. These events follow a different clinical pathway than live births, with distinct etiologies and limited availability of high-quality medication data. Moreover, early pregnancy exposures may be critical in understanding the effects of maternal medications, as some drugs could influence embryo viability or early gestational development. Excluding spontaneous abortions without discussion leaves an important evidence gap, be associated with modestly increased risks of early pregnancy loss.
4.Paternal Preconception Medications Use
Given the potential impact of paternal health and medication exposure on pregnancy and neonatal outcomes, the manuscript currently lacks information on this limitation. Emerging evidence suggests that paternal exposures prior to conception—such as certain medications, lifestyle factors, or environmental toxins—can influence sperm quality, epigenetic modifications, and early embryonic development, which may in turn affect pregnancy outcomes and neonatal health. The authors should explicitly acknowledge this gap, discuss how the absence of paternal medication data may influence the interpretation of maternal medication associations, and consider the potential implications for the study's conclusions.

(Remarks on code availability)
NA

Reviewer #3

(Remarks to the Author)
* Summary of the Manuscript:
The manuscript introduces PregMedNet, a large-scale analytic platform designed to evaluate the effects of maternal medication use during pregnancy on neonatal complications. Using 1,190,257 mother-baby dyads collected from a nationwide U.S. claims database, the study systematically analyzes 27,648 medication–disease pairs, reports odds ratios and adjusted odds ratios, and evaluates maternal drug-drug interactions (DDIs). A novel aspect of the work is the in vivo experimental validation of one association (maternal ondansetron to increased risk of neonatal bronchopulmonary dysplasia), and an exploration of biological mechanisms through a knowledge graph and graph-learning approach. The results are made accessible through an interactive website (https://pregmednet.stanford.edu/).

* Strengths:
- Very large and comprehensive dataset (over 1M dyads across multiple U.S. institutions), enabling statistical power and broad coverage of maternal medications and neonatal outcomes.

- Multifaceted methodology: statistical estimation of OR/aOR, systematic DDI analysis, experimental validation in mice, and knowledge-graph-based MoA hypotheses.

- Strong translational potential, providing a scalable platform and public website that can inform clinical guidance and future mechanistic research.

* Weaknesses / Recommendations:
- Interpretation vs. causality: while confounding challenges are acknowledged, some statements in the Results and Discussion read close to causal implications. Reinforcing non-causal language throughout would prevent over-interpretation.

- Several important demographic or behavioral variables (e.g., race, smoking, BMI, alcohol, socioeconomic status) are unavailable in claims data, which could lead to residual confounding. A table summarizing unavailable variables and how they may bias outcomes would increase transparency.

- The in vivo experiment is powerful, but covers only one association. The text should clarify that this serves as proof-of-concept, rather than full validation of the platform.

- The manuscript appropriately acknowledges that rare medications and uncommon drug combinations have reduced statistical power, which may prevent detection of significant associations even if an effect exists. One optional improvement would be to help readers understand the scale by briefly indicating how cohort sizes influence OR/DDI detectability. For example, by reporting the minimum dyad count among significant associations.

- The mechanistic (knowledge-graph) analysis is intriguing but exploratory. A clearer description of how shortest-path hypotheses could guide downstream biological experiments would strengthen scientific impact.

- Given the rapid progress in graph representation learning and graph neural networks (e.g., message passing, graph attention, heterogeneous graph transformers), the MoA discovery module feels underdeveloped relative to current state of the art. Even a short discussion comparing the chosen approach with more expressive GNN-based alternatives, or a brief justification for prioritizing shortest-path inference, would clarify the methodological choices and situate the contribution within the broader graph-learning landscape.

(Remarks on code availability)
I have reviewed the code on GitHub repository. The repository seems to be well-organized. It also contains a README for instructions.

Version 1:

Reviewer comments:

Reviewer #1

(Remarks to the Author)
I thank the authors for the rebuttal letter. They have addressed all questions and remarks regarding the in vivo experiment in detail and provided relevant answers and improved the methods and analysis in the manuscript and added further information in order to clarify their choices. I hereby, declare that I have no further questions regarding the in vivo experiments performed in this study and find the provided answers sufficient.

(Remarks on code availability)
NA

Reviewer #2

(Remarks to the Author)
Dear editor,
Dear authors,

Thank you for the opportunity to review the revised version of the manuscript.

I have considered the changes made in response to the previous review. The issues raised have been thoroughly addressed, and the revisions have significantly improved the clarity and quality of the paper.

I have no additional comments at this time.

(Remarks on code availability)
Dear editor,
Dear authors,

Thank you for the opportunity to review the revised version of the manuscript.

I have considered the changes made in response to the previous review. The issues raised have been thoroughly addressed, and the revisions have significantly improved the clarity and quality of the paper.

I have no additional comments at this time.

Reviewer #3

(Remarks to the Author)
I would like to thank the authors for addressing my concerns and questions, as well as improving the manuscript based on my suggestions. I have no more comments at this stage.

(Remarks on code availability)
I have reviewed the README and the structure of the repository. It seems to be well-documented and well-structured.

Open Access This Peer Review File is licensed under a Creative Commons Attribution 4.0 International License, which permits use, sharing, adaptation, distribution and reproduction in any medium or format, as long as you give appropriate credit to the original author(s) and the source, provide a link to the Creative Commons license, and indicate if changes were made.
In cases where reviewers are anonymous, credit should be given to 'Anonymous Referee' and the source.
The images or other third party material in this Peer Review File are included in the article's Creative Commons license, unless indicated otherwise in a credit line to the material. If material is not included in the article's Creative Commons license and your intended use is not permitted by statutory regulation or exceeds the permitted use, you will need to obtain permission directly from the copyright holder.
To view a copy of this license, visit https://creativecommons.org/licenses/by/4.0/

Reviewer #1 (Remarks to the Author):

The manuscript provides a nice tool for predicting the maternal-neonatal medication toxicity and can contribute to advancing maternal medication safety.
Based on the results of their proposed model, the researchers have found a link between ondansetron and increased neonatal respiratory risks. To validate this outcome, the researchers have conducted an in vivo mice model. However, there are a few concerns regarding the in vivo experimental design and data analysis. The experimental design is not fully explained (e.g. statistical calculation for sample size) and a justification of dosing/timing is missing:

We thank the reviewer for their positive comments and constructive feedback, which have helped improve the manuscript. Point by point responses are provided below.

1- According to PregMedNet results, researchers have found significant links between orally administered ondansetron and neonatal respiratory complications. However, in their in vivo experiment, they chose to administer this drug intraperitoneally. What was the justification for choosing IP rout instead of oral gavage?

We appreciate the opportunity to clarify the rationale for our experimental design. Ondansetron can be administered to human patients by oral or systemic (i.v.) route. For the animal experiments presented, we chose systemic, intraperitoneal administration as it can be done quickly in mice with minimal distress to pregnant dams. Giving ondansetron intraperitoneally also allowed us to ensure more consistent dosing and bioavailability compared to oral administration.

We have added the following text to the Method section (section 4.b.):

> "...Ondansetron can be administered to human patients via oral or systemic (intravenous) routes. For this study, we employed intraperitoneal injection to ensure precise dosing and bioavailability while minimizing handling stress for the pregnant dams…"

2- The average recommended dose of ondansetron in humans is 8 mg (not to exceed 16 mg). However, the researchers have used 5mg/kg b.w. in mice, which is relatively a higher dose compared to what is used in humans (ranging between 0.2 – 0.05 mg/kg b.w.). How do you justify the dose you chose?

We thank the reviewer's insightful comment, which allowed us to further elaborate on the justification for our experimental approach. Current dosing guidelines for hyperemesis gravidarum is 8 mg 3-4 times daily.[1,2] To convert the equivalent dosage to mice, we followed published guidelines for converting human to animal dosing taking into account differences in body surface area and relative differences in K_m[3]. Using these guidelines, 5 mg/kg daily dosing in mice is equivalent to 24 mg/day in a 60 kg human. In addition, multiple published studies using ondansetron in mice have used ranges of 0.1-25 mg/kg dosing [4–6]. Future investigations

could include pharmacokinetic studies of ondansetron and its metabolites in fetal and newborn tissues following maternal exposure.

The following Text have been added to the Methods section (section 4.b.):

> "...The dose of 5mg/kg body weight was determined based on clinical dosing guidelines for hyperemesis gravidarum (8mg, 3-4 times daily).[1] Human-to-mouse dose conversion was performed using guidelines taking into account differences in body surface area (BSA) and K_m.[3] The 5 mg/kg dose in mice corresponds to an approximately 24 mg/day regimen for a 60 kg human. This dose also falls within the range (0.1-25 mg/kg) previously reported in murine studies[4–6]…"

3- Ondansetron is used to prevent nausea and vomiting caused by chemotherapies or certain surgeries. Aside from that, it might be used for severe pregnancy nausea during the first trimester. However, researchers observed a link between ondansetron administration during the last trimester and neonatal complications. Therefore, the prescription of this medication to this group of pregnant patients could be accompanied by other underlying diseases or in combination to other medications. Considering these information:
a. Is the proposed PregMedNet tool capable of incorporate the additional parameters, such as underlying medical conditions and multidrug administration, in this prediction?
b. Ondansetron is usually prescribed as a single prophylactic dose, or for maximum 1-2 days post chemotherapy or surgery. However, in the in vivo experiment researchers used daily IP injections for 5-6 days. How do you justify your exposure duration? (There are of course a few off-label dosages for pruritus treatment that longer administration is recommended. However, the duration of medication is not specified in PregMedNet results)

a. We thank the reviewer's attention to this point. PregMedNet accounts for the complexity of maternal health by incorporating 68 maternal conditions and key demographic variables (e.g., maternal age, gestational age, and geographic location) (Methods 1.b., Supplementary Table 9). These parameters are utilized to calculate adjusted odds ratios (aORs), ensuring that the predicted neonatal outcomes are not biased by potential confounders. Furthermore, PregMedNet provides drug-drug interaction (DDI) that systematically evaluates the combined effects of two maternal medications administration, identifying potential synergistic risks to the neonate that may not be apparent from single-drug data (Results section: Systematic Analysis of maternal Drug-Drug Interactions).

b. We thank the reviewer for the opportunity to clarify the rationale for the experimental design. Ondansetron is commonly given for longer courses in the settings of post-chemotherapy nausea, hyperemesis gravidarum [2], cyclic vomiting, and other off-label uses in real world settings. Our experiments tested the potential link between ondansetron and abnormal lung morphogenesis like that seen in Bronchopulmonary Dysplasia (BPD). In these initial experiments, we therefore exposed pregnant mice at stages of fetal development where lung morphogenesis could be susceptible.

The following text has been added to the Methods section (4.b.):

"...To evaluate the impact on fetal lung morphogenesis, a 5–6 day dosing regimen was implemented. This duration captures critical developmental windows and remains within the scope of clinical guidelines for severe hyperemesis gravidarum, where extended treatment is clinically indicated.[2]"

4- Was there any effect of ondansetron on the course of pregnancy, e.g. number of pups per litter or resorptions, other (clinical) symptoms in pregnant mice etc?

We appreciate the reviewer's attention to this point. Control dams received intraperitoneal saline injections similarly to the ondansetron treated group. There were no statistically significant differences in reabsorption, litter size, pup weight, and pup survival. Additionally, pregnant dams dosed with ondansetron showed no clinical signs of morbidity. Figure 5.b. and 5.c. show that there are no differences of survival and weight gain between the two groups, factors that could otherwise exert secondary influences on lung development.

We have added the following text in the Results section (Maternal ondansetron impairs neonatal lung development *in vivo*):

"...There were no statistically significant differences in reabsorption or litter size, and pregnant dams dosed with ondansetron showed no clinical signs of morbidity. ..."

5- How was the sample size for this experiment calculated? Did you base the calculations on the number of pregnant mice or the pups? Since you collected samples from pups before weaning, did you consider each litter as an experimental unit, instead of individual pups? You had 20-24 pups from 4-5 litters, but you chose 9-12 mice for quantitative analysis. What was your criteria of choosing these mice? Was it a randomized selection, or did you select an average number of pups per litter? Regarding the statistical analysis performed in figure 5e, was it performed using all data points (135-180 images) or only on the average values per mouse?

For comparing lung morphogenesis in 11-day old mice, each newborn mouse was considered an individual experimental unit. Pups were randomly selected from each litter. Quality control ensured that only lungs properly inflated during fixation without obvious, gross damage to lung tissue were processed and analyzed. All sample processing, staining, imaging, and analysis was performed in a blinded manner.

Statistical analysis for Figure 5e was performed using a *t* test on all data points (135-180 images) to accurately assess the normal variation that occurs throughout regions of the lungs.

We have updated the following text in the Methods sections (4.c. & 4.e.):

Methods 4.c.: “On P11, pups were euthanized and the lungs were inflated with 4% PFA (Electron Microscopy Sciences 15713). For comparing lung morphogenesis, pups were randomly selected from each litter, with each newborn mouse considered an individual experimental unit. Following inflation, the lungs were collected and fixed in 4% PFA for 1 hour at room temperature. Quality control ensured that only lungs properly inflated during fixation without obvious, gross damage to lung tissue were processed and analyzed. ”

Methods 4.e.: “All sample processing, staining, imaging, and analysis was performed in a blinded manner. For quantification, brightfield images were captured with the Leica Thunder microscope using the 20X objective. Images were quantified on FIJI using the Mean Linear Intercept plug-in [7]. The image analysis parameter was set to 500 pixels between lines, and both horizontal and vertical chords were measured. All horizontal and vertical chords were averaged to get an image average value. For each pup, 3 representative lung sections were stained and imaged, with 5 images taken from each section for a total of 15 images quantified. Statistical analysis for Figure 5e was performed using a *t* test on all data points to accurately assess the normal variation that occurs throughout regions of the lungs.”

Reviewer #1 (Remarks on code availability):

Evaluating the code is not within my expertise.

Reviewer #2 (Remarks to the Author):

Strengths
1.Clear Public Health Relevance and Timeliness

PregMedNet addresses a critical and growing public health need: the lack of robust evidence on the safety of medications used during pregnancy, particularly with respect to long-term outcomes. By integrating a wide range of high-quality data sources—including hospital records and claims data—the platform directly responds to urgent challenges in maternal and neonatal health. In addition, PregMedNet enables finer-grained investigation of maternal medication effects by retrieving drug information in a data-driven, compound-level manner and by distinguishing between routes of administration. This distinction allows the system to differentiate clinically distinct use cases for the same medication, such as topical betamethasone for skin inflammation versus intramuscular betamethasone for preventing preterm labor complications. Finally, the study examines the additive effects of concurrent maternal medication exposures on neonatal outcomes through a retrospective drug–drug interaction (DDI) analysis. To our knowledge, this represents the first large-scale, real-world, multi-institutional effort to systematically screen the effects of maternal drug combinations on neonatal complications.
2.Methodological Innovation

This project introduces several important methodological advances: (a) a systematic approach for identifying associations between maternal medication use and neonatal complications, (b) robust adjustment for confounding variables across numerous medication–disease pairs, (c) in vivo experimental validation supporting one of the data-driven hypotheses, and (d) the proposal of plausible mechanisms of action (MoA). A central contribution of this work is the implementation of a machine-learning–based framework for confounding adjustment

We thank the reviewers for the positive comments and the constructive feedback on the manuscript, which we address in detail below.

Points for considerations

1.Clarification of Twins and Other Multiple Births

The manuscript would benefit from a clearer justification for the exclusion of twins and other multiple births. Multiple gestations inherently carry higher and clinically distinct risks, that are not directly comparable to singleton pregnancies. The PregMedNet by it inherent approach could help close the gap of medications effects on multiple births.

Thank you for raising this important point. We agree that the higher and clinically distinct risks associated with multiple gestations distinguish them from singleton pregnancies. However, multiple gestations introduce confounding due to the influence of shared intrauterine factors, which can complicate the attribution of neonatal outcomes to maternal medication effects, leading us to restrict our analysis to live singleton births.
In particular, we adjusted for various maternal comorbidities when estimating adjusted odds ratios to further minimize bias and isolate the specific effects of maternal medication on neonatal complications, while providing raw odds ratios together. This approach aligns with our primary objective of identifying perinatal medication effects on neonatal complications.
While we have focused on live singleton births in this study, we agree that future extensions of PregMedNet to multiple gestations could help elucidate the maternal medication effects in these clinically distinct pregnancies.

We have added the following text to the Discussion section (paragraph 10) to clarify this point:

> "The cohort definition adopted in this study entails several limitations. We restricted the cohort to **singleton**, first pregnancy live births to better isolate the impact of maternal medication on neonatal outcomes, while **minimizing potential confounding from multiple gestations**, multiparity, and spontaneous abortion. **Multiple gestations** and multiparity **introduce shared intrauterine factors and intra-maternal correlations that are difficult to fully disentangle**…"

2.First Pregnancy

Similarly, the decision to include only the first pregnancy per woman deserves further explanation.

We restricted the analysis to the first pregnancy per woman to avoid intra-maternal correlation across parities and to preserve the statistical independence of observations. Subsequent pregnancies may be influenced by prior pregnancy outcomes and evolving maternal comorbidities, which could introduce additional confounding that is difficult to fully disentangle. By focusing on the first pregnancy, we aimed to isolate maternal medication-associated effects on neonatal complications while reducing potential structural confounding. Future extensions of PregMedNet to include multiple parities would help delineate the distinct impacts of birth order.

Consistent with our previous point, the following clarification was added to the Discussion (paragraph 10):

> "...We restricted the cohort to singleton, **first pregnancy** live births **to better isolate the impact of maternal medication on neonatal outcomes, while minimizing potential confounding from** multiple gestations, **multiparity**, and spontaneous abortion. Multiple gestations and **multiparity introduce shared intrauterine factors and intra-maternal correlations that are difficult to fully disentangle**…"

3.Spontaneous abortion

The exclusion of spontaneous abortions also warrants additional justification. These events follow a different clinical pathway than live births, with distinct etiologies and limited availability of high-quality medication data. Moreover, early pregnancy exposures may be critical in understanding the effects of maternal medications, as some drugs could influence embryo viability or early gestational development. Excluding spontaneous abortions without discussion leaves an important evidence gap, be associated with modestly increased risks of early pregnancy loss.

We acknowledge the reviewer's point that excluding spontaneous abortions may limit a holistic assessment of how maternal medications influence pregnancy outcomes. However, this study was intentionally designed to focus on live births in order to more specifically estimate maternal medication-associated effects on neonatal outcomes. Early pregnancy loss is strongly influenced by genetic factors, with approximately 50% percent of cases attributable to fetal chromosomal abnormalities.[8] By restricting our cohort to the live births, we aimed to minimize the impact of these substantial and often unmeasured genetic confounders which are frequently underreported and difficult to identify in clinical dataset. Future research incorporating more granular data on early pregnancy loss is certainly warranted to provide a more comprehensive view of these medications' reproductive safety profile.

We have added the following explanation to the Discussion section (paragraph 10), acknowledging this as a limitation:

> "...We restricted the cohort to singleton, first regnancy **live births** to better isolate the impact of maternal medication on neonatal outcomes, **while minimizing potential confounding from** multiple gestations, multiparity, and **spontaneous abortion**… **Furthermore, including spontaneous abortions would introduce significant genetic confounding, as approximately half of early pregnancy loss is due to**

chromosomal abnormalities—factors that are frequently underreported and challenging to classify in clinical datasets[8] …"

4.Paternal Preconception Medications Use

Given the potential impact of paternal health and medication exposure on pregnancy and neonatal outcomes, the manuscript currently lacks information on this limitation. Emerging evidence suggests that paternal exposures prior to conception—such as certain medications, lifestyle factors, or environmental toxins—can influence sperm quality, epigenetic modifications, and early embryonic development, which may in turn affect pregnancy outcomes and neonatal health. The authors should explicitly acknowledge this gap, discuss how the absence of paternal medication data may influence the interpretation of maternal medication associations, and consider the potential implications for the study's conclusions.

We thank the reviewer for highlighting the importance of paternal health and medication impacts on neonatal outcomes.[9,10] We have now acknowledged this in the Discussion section (paragraph 10), noting that paternal exposures may influence neonatal outcomes and early embryonic development. We explained that the absence of paternal data may introduce residual confounding, which warrants caution in interpretation and highlights the need for future research to utilize biparental datasets.

The following was added to the Discussion (paragraph 10):

> "...Additionally, the potential influence of paternal comorbidities and medication exposure on neonatal complications should be considered, as emerging evidence suggests that paternal health conditions and pharmacologic exposures may influence offspring outcomes.[9,10]…"

For completeness, the full consolidated paragraph incorporating these points is provided below (Discussion, paragraph 10):

> "The cohort definition adopted in this study entails several limitations. We restricted the cohort to singleton, first pregnancy live births to better isolate the impact of maternal medication on neonatal outcomes, while minimizing potential confounding from multiple gestations, multiparity, and spontaneous abortion. Multiple gestations and multiparity introduce shared intrauterine factors and intra-maternal correlations that are difficult to fully disentangle. Furthermore, including spontaneous abortions would introduce significant genetic confounding, as approximately half of early pregnancy loss is due to chromosomal abnormalities [8] —factors that are frequently underreported and challenging to classify in clinical datasets. While these exclusion criteria ensure high internal validity for our primary analysis, they may limit the generalizability of the findings to more complex pregnancies. Therefore, the impact of maternal medication in these specific subgroups—such as multiple gestations or cases involving early pregnancy loss—remains an important area for further investigation. Additionally, the potential influence of paternal comorbidities and medication exposure on neonatal complications

should be considered, as emerging evidence suggests that paternal health conditions and pharmacologic exposures may influence offspring outcomes.[9,10] Future research incorporating more granular, linked clinical data that can address these limitations is warranted to establish a truly comprehensive reproductive safety profile for these medications."

Reviewer #2 (Remarks on code availability):

NA

Reviewer #3 (Remarks to the Author):

* Summary of the Manuscript:
The manuscript introduces PregMedNet, a large-scale analytic platform designed to evaluate the effects of maternal medication use during pregnancy on neonatal complications. Using 1,190,257 mother-baby dyads collected from a nationwide U.S. claims database, the study systematically analyzes 27,648 medication–disease pairs, reports odds ratios and adjusted odds ratios, and evaluates maternal drug-drug interactions (DDIs). A novel aspect of the work is the in vivo experimental validation of one association (maternal ondansetron to increased risk of neonatal bronchopulmonary dysplasia), and an exploration of biological mechanisms through a knowledge graph and graph-learning approach. The results are made accessible through an interactive website (https://pregmednet.stanford.edu/).

* Strengths:
- Very large and comprehensive dataset (over 1M dyads across multiple U.S. institutions), enabling statistical power and broad coverage of maternal medications and neonatal outcomes.

- Multifaceted methodology: statistical estimation of OR/aOR, systematic DDI analysis, experimental validation in mice, and knowledge-graph-based MoA hypotheses.

- Strong translational potential, providing a scalable platform and public website that can inform clinical guidance and future mechanistic research.

We thank the reviewer for the positive comments and the constructive feedback to improve the manuscript, which we address in further detail below.

* Weaknesses / Recommendations:
- Interpretation vs. causality: while confounding challenges are acknowledged, some statements in the Results and Discussion read close to causal implications. Reinforcing non-causal language throughout would prevent over-interpretation.

We sincerely thank the reviewer for this important point. We agree that maintaining a clear distinction between statistical association and causal implication is crucial in large-scale observational studies. As suggested, we have carefully revised the manuscript to reinforce non-causal language throughout, particularly in the Results and Discussion sections, to prevent any over-interpretation of our findings.
Examples of these revisions include:

- Replacing causal terminology such as "increasing/decreasing risks" with "increased/decreased risk associations";
- Reframing "benefits of maternal medications" as "reduced risk associations between medications and neonatal complications";
- Revising interpretations of negative/positive β_3 values from "reducing/increasing risk" to "indicating a downward/upward shifts in the odds associated with interaction"
- Replace "confirms/reconfirms previously known associations" with "aligned with previously reported clinical findings"

These changes are highlighted in blue in the revised manuscript.

- Several important demographic or behavioral variables (e.g., race, smoking, BMI, alcohol, socioeconomic status) are unavailable in claims data, which could lead to residual confounding. A table summarizing unavailable variables and how they may bias outcomes would increase transparency.

We have added **Supplementary Table 11**, which systematically summarizes key demographic and behavioral variables unavailable in claims data, along with their potential impact on neonatal outcomes and the likely direction of bias in association estimates. We believe this addition improves transparency regarding sources of residual confounding inherent to claims-based analyses.

Supplementary Table 11. Unavailable demographic and behavioral variables and their impact on association estimates

Unavailable Variable	Potential Impact on Neonatal Outcomes	Likely Direction of Bias
Maternal Race/Ethnicity	Baseline risk differences and healthcare access disparities	Uncertain
Smoking habitus	Confounding in respiratory and growth-related outcomes	Likely overestimation
Alcohol use	Neurodevelopmental and growth-related confounding	Likely overestimation
Weight/Height (BMI)	Confounding in metabolic and gestational complications	Likely overestimation
Socioeconomic status (SES)	Differences in care access and environmental exposures, nutritional status, and comorbidity burden	Uncertain

Medication adherence	Exposure misclassificaiton (prescription issued ≠ drug ingestion)	Toward null

- The in vivo experiment is powerful, but covers only one association. The text should clarify that this serves as proof-of-concept, rather than full validation of the platform.

We appreciate the reviewer's constructive feedback regarding the scope of our in vivo validation. We agree that the previous description of the experimental support could be misinterpreted as implying full validation of the platform. We have now clarified throughout the manuscript that the *in vivo* experiment is intended as a proof-of-concept, illustrating PregMedNet's hypothesis-generating capacity and its ability to prioritize biologically plausible associations from large-scale claims data, rather than serving as comprehensive validation. Specifically, we revised the Discussion (paragraph 7) to explicitly state this distinction.

The revised text is provided below:

> "As such, this *in vivo* experiment serves as a proof-of-concept, demonstrating PregMedNet's capacity to generate biologically plausible hypotheses from large-scale, claims data. While previous research studies have utilized claims databases to investigate maternal medication safety, this study, to the best of our knowledge, represents the first attempt to experimentally evaluate and provide preliminary support for such hypotheses."

- The manuscript appropriately acknowledges that rare medications and uncommon drug combinations have reduced statistical power, which may prevent detection of significant associations even if an effect exists. One optional improvement would be to help readers understand the scale by briefly indicating how cohort sizes influence OR/DDI detectability. For example, by reporting the minimum dyad count among significant associations.

We agree that clarifying the relationship between cohort size and the detectability of OR/DDI associations is crucial for understanding the statistical scale and reliability of our findings. As suggested, we have added a detailed analysis of exposure case distributions to the Discussion section (14th paragraph) to contextualize these power-related limitations.

Specifically, we report that significant associations were identified with a minimum dyad count of 11 exposure cases for Odds Ratios (ORs, aORs) and 12 for Drug-Drug-Disease Interactions (DDIs). This threshold is consistent with the CMS Limited Data Set (LDS) Data Use Agreement (DUA),[11] which prohibits reporting results derived from cohorts with fewer than 11 patients to protect patient privacy.

The data show that while PregMedNet can identify risks even in small cohorts, the median number of exposure cases for significant findings was 81 (interquartile range [IQR]: 30–277.75) for raw ORs and 656 (IQR: 325–1,545) for adjusted ORs. This shift from 81 to 656 indicates that the adjustment process inherently requires more robust evidence and larger sample sizes to maintain statistical significance while controlling for confounders. We believe this addition provides readers with the necessary context to evaluate the robustness of our results and explains why associations involving rare medications may remain undetectable due to statistical and privacy constraints.

The following text and figure (Supplementary figure 10) have been added to the Discussion section (paragraph 14):

> “To further contextualize these power-related limitations, we report that significant associations were identified with a minimum dyad count of 11 exposure cases for Odds Ratios (ORs, aORs) and 12 for Drug-Drug-Disease Interactions (DDIs). This is consistent with the CMS Limited Data Set (LDS) Data Use Agreement (DUA),[11] which restricts the reporting of results derived from cohorts with fewer than 11 cases to protect patient privacy. This indicates that while PregMedNet is capable of identifying risks even within small cohorts near the reporting threshold, associations involving rarely prescribed medications or uncommon drug combinations may fall below this limit, remaining undetectable or unreported due to statistical and privacy constraints. Among the significant findings, the median number of exposure cases was 81 (IQR: 30–277.75) for raw ORs, 656 (IQR: 325–1,545) for adjusted ORs, and 35 (IQR: 34–45) for DDIs. The distribution of the number of exposure cases for ORs and aORs are depicted in Supplementary Figure 10. The shift of the median exposure count from 81 to 656 after adjustment suggests that only associations with a larger number of exposures retained statistical significance after controlling for confounders, as the adjustment process inherently requires more robust evidence to overcome potential bias. Taken together, while acknowledging these inherent constraints regarding rarely used medications, our findings provide a robust overview of perinatal medication risks, with reported associations interpreted within the context of exposure prevalence, statistical power, and reporting thresholds.”

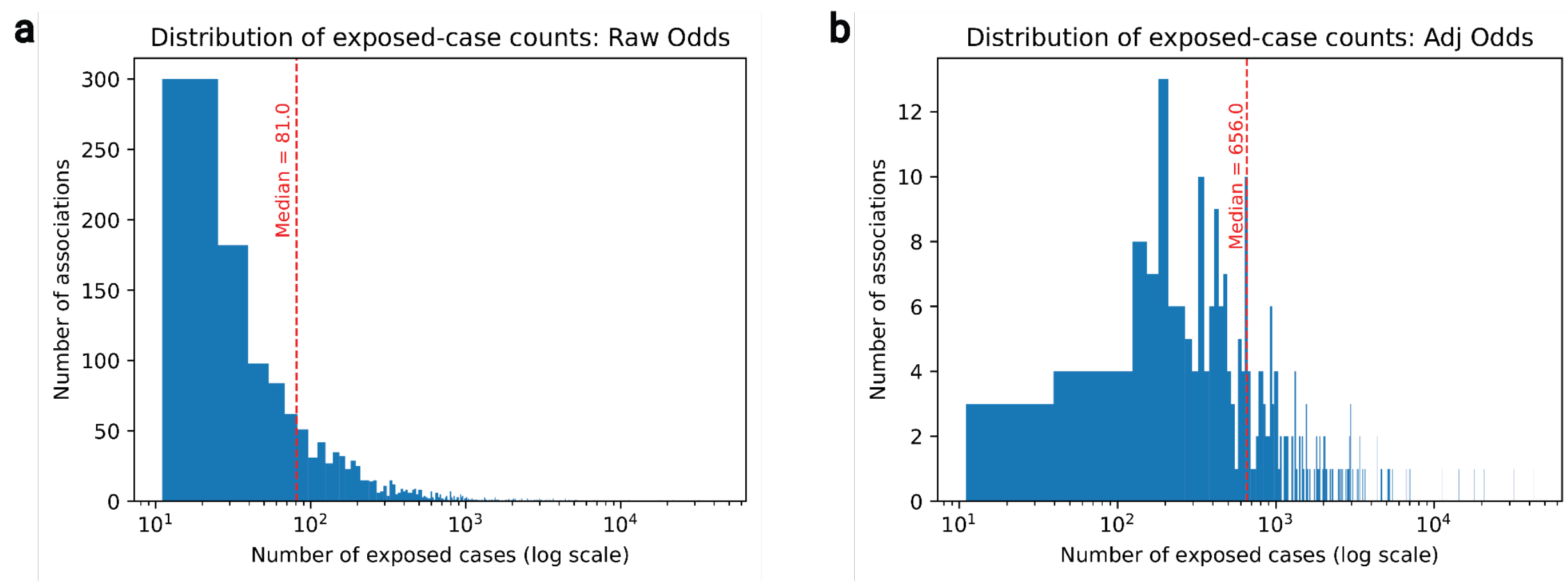

Supplementary Figure 10. Distributions of exposed-case counts for signification associations. Histograms showing the distribution of the number of exposed cases among significant associations for (a) raw odds ratios (ORs) and (b) adjusted odds ratios (aORs). The x-axis represents the number of exposed cases on a logarithmic scale, and the y-axis represents the number of associations. Red dashed lines indicate the median number of exposed cases for each analysis.

- The mechanistic (knowledge-graph) analysis is intriguing but exploratory. A clearer description of how shortest-path hypotheses could guide downstream biological experiments would strengthen scientific impact.

We thank the reviewer for this insightful comment. We agree that the knowledge-graph (KG) analysis, while exploratory, gains significant scientific impact when it provides a clear roadmap for downstream validation. In the revised manuscript, we have clarified that our shortest-path approach is grounded in the principle of parsimony,[12] aiming to identify the most biologically plausible and direct connections between medications and outcomes.

We have specifically highlighted how these identified paths serve as a hypothesis-prioritization tool by reducing the vast biological search space into a minimal set of candidate nodes (e.g., specific proteins or pathways) for targeted experimental analysis. Furthermore, we address the choice of a transparent shortest-path method over complex graph machine learning to avoid unintended artifacts from binary network topology, ensuring that our MoA hypotheses remain interpretable and clinically grounded. These details have been added to the Discussion section (paragraph 8) to better articulate the downstream utility of our KG analysis.

The following text has been added to the Discussion section (paragraph 8):

> "...A knowledge graph aggregates a wide range of experimentally supported molecular and biological interactions into a unified network, enabling a holistic view of biological relationships at a systems level. For significant medication-outcome associations lacking direct known interactions, we assume potential biological connectivity through

intermediate entities and apply shortest-path analysis to identify the most direct biological routes. Grounded in the principle of parsimony,[12] this approach prioritizes shortest paths as the most biologically plausible connections. While exploratory, these shortest paths serve as a hypothesis-prioritization tool, highlighting a minimal set of candidate nodes for downstream experimental analysis. …"

- Given the rapid progress in graph representation learning and graph neural networks (e.g., message passing, graph attention, heterogeneous graph transformers), the MoA discovery module feels underdeveloped relative to current state of the art. Even a short discussion comparing the chosen approach with more expressive GNN-based alternatives, or a brief justification for prioritizing shortest-path inference, would clarify the methodological choices and situate the contribution within the broader graph-learning landscape.

We appreciate the reviewer's insightful suggestion regarding the use of advanced graph representation learning. We agree that GNN-based methods (e.g., message passing, GAT, or HGT) are powerful tools in many contexts. However, for the MoA discovery module in this study, we intentionally prioritized a transparent, shortest-path-based approach over complex GNN alternatives for several methodological and biological reasons.

First, our current knowledge graph aggregates experimentally verified biological interactions in a binary setting, lacking granular quantitative metadata such as interaction strength, directionality, or cell-specific context. Under these constraints, highly expressive GNNs can be prone to learning network topology artifacts—such as node centrality or hubness—rather than true biological interaction strengths. Second, MoA discovery for clinical applications requires a traceable and interpretable causal chain. The recursive nature of GNN message-passing can obscure specific intermediate nodes, whereas shortest-path inference provides an explicit roadmap of intermediate proteins and pathways that can be directly inspected and experimentally validated.

By adhering to the principle of parsimony, our approach minimizes computational complexity while yielding actionable biological hypotheses. We have added a discussion of these trade-offs to the Discussion section (paragraph 15) and outlined how future extensions of PregMedNet could incorporate granular metadata and advanced GNN-based re-ranking as such data become available.

The following text have been added to the discussion section (paragraph 15):

"Finally, while the knowledge graph used for MoA discovery aggregates comprehensive and verified biological associations, it currently exists in a binary setting, lacking granular information regarding the relative strength, directionality, and cell-specific context of these interactions. In this context, applying highly expressive graph machine learning may yield representations driven by network topology—such as node centrality or hubness—rather than real-world biological strength, which could introduce unintended artifacts. Therefore, we utilized a transparent, shortest-path-based approach for MoA

hypothesis generation to maintain parsimony and interpretability. Future extensions of PregMedNet incorporating granular metadata, coupled with the application of advanced graph-based machine learning, may potentially further enhance the predictive power and precision of MoA-based hypothesis generation."

Reviewer #3 (Remarks on code availability):

I have reviewed the code on GitHub repository. The repository seems to be well-organized. It also contains a README for instructions.

References

1. The Management of Nausea and Vomiting of Pregnancy and Hyperemesis Gravidarum.
2. Slattery, J. *et al.* Ondansetron use in nausea and vomiting during pregnancy: A descriptive analysis of prescription patterns and patient characteristics in UK general practice. *Br. J. Clin. Pharmacol.* **88**, 4526–4539 (2022).
3. Reagan-Shaw, S., Nihal, M. & Ahmad, N. Dose translation from animal to human studies revisited. *FASEB J.* **22**, 659–661 (2008).
4. Wu, M., Cheng, Z., Le, A. T., Tan, Y. & Peltz, G. Optimizing a therapy for opiate use disorders: Characterizing ondansetron pharmacokinetics in blood and brain. *Clin. Transl. Sci.* **16**, 216–223 (2023).
5. Erhan, E. *et al.* Ondansetron does not block tramadol-induced analgesia in mice. *Methods Find. Exp. Clin. Pharmacol.* **27**, 629–632 (2005).
6. Wildeboer, K. M., Zheng, L., Choo, K. S. & Stevens, K. E. Ondansetron results in improved auditory gating in DBA/2 mice through a cholinergic mechanism. *Brain Res.* **1300**, 41–50 (2009).
7. Crowley, G. *et al.* Quantitative lung morphology: semi-automated measurement of mean linear intercept. *BMC Pulm. Med.* **19**, 206 (2019).
8. American College of Obstetricians and Gynecologists' Committee on Practice Bulletins—

Gynecology. ACOG practice bulletin no. 200: early pregnancy loss. *Obstet. Gynecol.* **132**, e197–e207 (2018).

9. Kasman, A. M. *et al.* Association of preconception paternal health on perinatal outcomes: analysis of U.S. claims data. *Fertil. Steril.* **113**, 947–954 (2020).

10. Zeng, Y., Lin, W. & Zhuang, W. Safety concerns of paternal drug exposure on fertility, pregnancy and offspring: An analysis based on the FDA adverse event reporting system. *Andrology* **13**, 1420–1431 (2025).

11. CMS.gov: Data Disclosures and Data Use Agreements (DUAs) - Limited Dataset (LDS) files. https://www.cms.gov/data-research/files-for-order/data-disclosures-and-data-use-agreements-duas/limited-data-set-lds.

12. Stewart, C. B. The powers and pitfalls of parsimony. *Nature* **361**, 603–607 (1993).
